# Discovering Synergistic Compounds with BYL-719 in PI3K Overactivated Basal-like PDXs

**DOI:** 10.3390/cancers15051582

**Published:** 2023-03-03

**Authors:** David C. Boyd, Emily K. Zboril, Amy L. Olex, Tess J. Leftwich, Nicole S. Hairr, Holly A. Byers, Aaron D. Valentine, Julia E. Altman, Mohammad A. Alzubi, Jacqueline M. Grible, Scott A. Turner, Andrea Ferreira-Gonzalez, Mikhail G. Dozmorov, J. Chuck Harrell

**Affiliations:** 1 Department of Pathology, Virginia Commonwealth University, Richmond, VA 23298, USA; 2 Integrative Life Sciences Program, Virginia Commonwealth University, Richmond, VA 23284, USA; 3 C. Kenneth and Dianne Wright Center for Clinical and Translational Research, Virginia Commonwealth University, Richmond, VA 23298, USA; 4 Department of Biostatistics, Virginia Commonwealth University, Richmond, VA 23219, USA; 5 Massey Cancer Center, Virginia Commonwealth University, Richmond, VA 23298, USA

**Keywords:** patient-derived xenograft, precision medicine, basal-like breast cancer, triple-negative breast cancer, synergism, BYL-719 (alpelisib), everolimus, dronedarone, afatinib

## Abstract

**Simple Summary:**

Basal-like breast cancers comprise the majority of triple-negative breast cancers (TNBC) and lack effective treatment options that have a sustained response. Part of the reason that they are hard to eliminate is that they exhibit high levels of genomic instability and cellular diversity. Most basal-like tumors have high levels of Phosphoinositide 3-Kinase (PI3K) pathway activity. A key driver of this pathway is PIK3CA. Many compounds have been made to target PIK3CA and have become standard-of-care in some estrogen-dependent patients; however, in TNBC patients, PI3K inhibitors (PI3Ki) as single agents thus far have shown limited duration at tolerable doses. The goal of this study was to identify and/or repurpose drugs that, when combined with PI3Ki, yield a significant inhibition of tumor growth. When treated in conjunction with the PI3Ki BYL-719, which is clinically prescribed as alpelisib, 20 potent drug combinations were identified and formed a basis toward clinical studies with these molecules.

**Abstract:**

Basal-like triple-negative breast cancer (TNBC) tumor cells are difficult to eliminate due to resistance mechanisms that promote survival. While this breast cancer subtype has low PIK3CA mutation rates when compared to estrogen receptor-positive (ER+) breast cancers, most basal-like TNBCs have an overactive PI3K pathway due to gene amplification or high gene expression. BYL-719 is a PIK3CA inhibitor that has been found to have low drug-drug interactions, which increases the likelihood that it could be useful for combinatorial therapy. Alpelisib (BYL-719) with fulvestrant was recently approved for treating ER+ breast cancer patients whose cancer had developed resistance to ER-targeting therapy. In these studies, a set of basal-like patient-derived xenograft (PDX) models was transcriptionally defined with bulk and single-cell RNA-sequencing and clinically actionable mutation profiles defined with Oncomine mutational profiling. This information was overlaid onto therapeutic drug screening results. BYL-719-based, synergistic two-drug combinations were identified with 20 different compounds, including everolimus, afatinib, and dronedarone, which were also found to be effective at minimizing tumor growth. These data support the use of these drug combinations towards cancers with activating PIK3CA mutations/gene amplifications or PTEN deficient/PI3K overactive pathways.

## 1. Introduction

### 1.1. Etiology of Triple Negative Breast Cancer in the US

In total, 300,590 new breast cancer diagnoses and 43,700 breast cancer-related deaths are predicted for 2023 [1], an increase from the year before [2]. Basal-like tumors have the most limited treatment options of the breast cancer intrinsic subtypes because they lack ER, PR, and HER2, which are susceptible to inhibition with anti-estrogens or HER2-targeted agents. They also have high rates of DNA mutations and amplifications, which increases the heterogeneity of cancer cells and can lead to more genetically diverse subpopulations [3]. Basal-like breast cancer patients have a low 5-year survival rate, partially because of their cancers’ tendency to metastasize to lung, bone, brain, and other organs [4]. Because of these challenges, the basal-like disease has a worse prognosis than breast cancer overall and is most in need of new effective therapeutic options.

### 1.2. Importance of PIK3CA in Breast Cancer

PIK3CA is the second most commonly aberrant gene in breast cancer, after TP53 [5]. PIK3CA codes for the catalytic subunit p110α that converts the lipid PIP2 into PIP3. This acts as a substrate for AKT and its activating kinases, which have oncogenic downstream effects [6]. PIK3CA often has an activating mutation that drives oncogenic transformation [7,8]. Overexpression of PIK3CA is also pathogenic: a gain of 1 or 2 copies is enough to see a significant increase in PI3K expression [5]. PIK3CA expression is often associated with resistance to therapeutics, such as EGFR inhibitors [9]. The loss of a regulatory protein of p110α, PTEN, often happens concurrently with PIK3CA mutations [10] but can increase PI3K pathway activity without PIK3CA alteration. PTEN can have normal CNV on the DNA level but be lost on the RNA level through gene silencing via methylation of the promoter [11]. Activating mutations of PIK3CA occur in basal-like tumors at a rate of approximately 10%, yet nearly all exhibit robust upregulation of the PI3K pathway. This central network is also activated through mutations or loss of PTEN or INPP4B or changes in RTKs [12] and AKT3. Collectively, PI3K is most highly expressed in basal-like breast cancers compared to other subtypes [13]. Despite the attention of researchers on PIK3CA, alpelisib (BYL-719) is the only p110α inhibitor that is FDA-indicated for use in breast cancer and only in conjunction with fulvestrant, an estrogen receptor antagonist [14]. This combination is only approved for patients with ER+ HER2- breast cancer that are male or post-menopausal female with PIK3CA mutant after disease progression after or while being treated with endocrine-based treatment [15]. A novel combination therapy approach with BYL-719 may provide pharmacokinetic synergism and improve meaningful clinical efficacy, such as disease-free survival, all while lessening the likelihood of severe adverse events through reduced drug exposure.

### 1.3. Patient-Derived Xenografts

PDXs (patient-derived xenografts) are similar to cancer cell lines but differ in that they are maintained in a physiological setting as soon as they are isolated from the patient and for subsequent passages. These models are valuable for preclinical trials because PDX models have been shown to closely match their patient counterparts [16,17], both in genomic profile and response to treatment [18]. In comparison, some cell lines have been shown to diverge from human patient tumors and lose intratumor heterogeneity and have alterations in protein levels revealed using histopathology [19]. PDX models have unique difficulties that cell lines do not have in establishing; one among them is establishment rates as low as 4% [20], as well as requiring mouse implantation instead of media-based tissue culture. Among other institutions, cohorts of human breast cancer PDXs have been created at Huntsman Cancer Institute [17] (HCI), Baylor College of Medicine [21] (BCM), University of Colorado, Denver [22] (UCD), and Washington University, St. Louis which developed the Washington Human in Mouse (WHIM) [23] PDXs.

### 1.4. Approach

BYL-719 is approved as part of a multi-drug treatment strategy for ER+ disease, but it is not curative as a single agent due to the development of drug insensitivity. To overcome resistance, an additional agent would be required. In this study, high throughput screening was utilized to identify synergistic candidates. Everolimus, an mTOR inhibitor, afatinib, an Epidermal Growth Factor Receptor (EGFR) inhibitor, and dronedarone, a multi-ion channel inhibitor, were selected and tested in vivo and in vitro in PI3K aberrant and PI3K overactive PDXs. All three combinations proved promising in PIK3CA aberrant basal-like breast cancer in the mouse models.

## 2. Materials and Methods

### 2.1. Cell Culture and Cell Lines

Cells were passaged in tissue culture-treated filter flasks in a 37 °C, 5% CO_2_ environment. To passage and in assays, the cells were grown in DMEM media with 10% FBS and 2% Pen/Strep for MDA-MB-453. Isogenic MCF10A wild type, E545K, and H1047R PIK3CA, kind gifts from Ben Ho Park [24] were utilized in a similar media that contained horse serum instead of FBS.

### 2.2. Targeted Mutation Profiling

To identify clinically actionable mutations targeted next-generation sequencing (NGS) was performed by VCU Pathology Molecular Diagnostics laboratory with clinically validated methodology used for diagnostic tumor profiling. PDX tumors were excised from mice, flash frozen, prepared into frozen sections using optimal cutting temperature (OTC) compound, and total nucleic acids extracted. NGS using the Oncomine Comprehensive Assay v3 (ThermoFisher, Waltham, MA, USA) was performed as previously described [25] to identify DNA mutations, DNA copy number variations, and RNA fusions across cancer-related genes.

### 2.3. PDXs and Passaging

The UCD52, WHIM30, HCI-001, HCI-010, and HCI-013 PDXs were utilized. PDXs were passaged in vivo by injecting single cell suspensions in a 1:1 ratio of HF (Hanks’ Balanced Salt Solution + 2% FBS) and Matrigel into the abdominal mammary fat pad of female NSG mice. At the end of a passage, the tumor-bearing mouse was euthanized, and its primary tumor or tumors were immediately excised and placed in PBS. The tumors were minced with a sterile razor blade and placed in tumor digestion solution (DMEM/F12 with 5% FBS, 0.0533 mg/mL hyaluronidase, and 2.4 mg/mL collagenase) on a thermoregulated tube cycler at 37 °C for 1 h. Solutions were centrifuged, and their supernatants were discarded. Pellets were resuspended in red blood cell lysis buffer, centrifuged, and again supernatants discarded into aspiration bleach traps. Pellets were resuspended in trypsin. Cells were resuspended at 500,000 cells per 100 μL HF; this was mixed 1:1 with Matrigel or Cultrex before injecting into the recipient mouse. Tumor area was calculated using volume = length times width.

### 2.4. Cell Culture Viability Assays

Cells were plated as described above for secondary cell lines or PDX single cell suspension in 96 or 384 well plates by hand or by automated robotic micropipette (Integra, Hudson, NH, USA, Assist Plus). PDX cells were plated in M87 media [26]. Drugs or control vehicles were pipetted into the wells immediately after plating for PDX single cell suspensions or after allowing cells to adhere, 2 h minimum for adherent cell lines. After the desired timepoint, a Cell Titer Glo luminescence assay was performed using a plate reader (BMG LABTECH, Ortenberg, Germany, POLARstar OPTIMA), and percent viability was calculated relative to vehicle control.

### 2.5. In Vivo Drug Trials and Mouse Observations

All in vivo studies were approved by VCU IACUC protocol approval through Animal Care and Use Program, an AAALAC-accredited program. BYL-719 (50 mg/kg) and afatinib (25 mg/kg) were administered via oral gavage (OG) in 100 μL of 1% methylcellulose 6 times a week. Everolimus (10 mg/kg) was administered via OG 3 times a week. Dronedarone (50 mg/kg) was administered via intraperitoneal (IP) injection in 100 μL of 10% DMSO, 40% PEG300, 5% Tween-80, and 45% Saline solution 6 times a week. Tumors were measured 3 times a week for health checks. Mouse weights were recorded once or twice a week. Mice that reduced in weight by 10% or reached maximum tumor burden by protocol guidelines were euthanized.

### 2.6. Bulk RNA Sequencing

Dry ice flash frozen tumors were processed using Qiagen Rneasy Kit in conjunction with QIAshredder tubes and RNA Zap. The quality of the sample was tested using Nanodrop. To construct the library, the RNA sample was first quantified with the Qubit 2.0 Fluorometer (ThermoFisher Scientific, Waltham, MA, USA) and checked for its integrity with TapeStation (Agilent Technologies, Santa Clara, CA, USA) Then, NEBNext Ultra II RNA Library Prep Kit for Illumina was used to prepare the RNA sequencing library according to manufacturer’s instructions (New England Biolabs, Ipswich, MA, USA). Afterward, the mRNAs were briefly enriched with Oligod(T) beads and then fragmented for 15 min at 94 °C. Subsequently, the first- and second-strand cDNA fragments were synthesized, after which their 3’ ends were end-repaired and adenylated. Following this, universal adapters were ligated to the cDNA fragments, and then index addition and library enrichment via PCR were performed with limited cycles. Finally, to validate the finished sequencing library, Agilent TapeStation (Agilent Technologies) was used, and the library was quantified with the Qubit 2.0 Fluorometer (ThermoFisher Scientific) as well as by quantitative PCR (KAPA Biosystems, Wilmington, MA, USA).

Sequencing was performed by Azenta. Sequencing libraries were first multiplexed and clustered onto a flow cell, after which the flow cell was inserted into the Illumina HiSeq 4000 instrument according to manufacturer’s instructions. A 2 × 150 bp paired-end (PE) configuration was selected to sequence the samples. The HiSeq Control Software (HCS) was utilized to conduct image analysis and base calling. Once the raw BCL data were generated, it was converted into FASTQ format and, lastly, demultiplexed with the Illumina BCL2FASTQ 2.17 program. For index sequence identification, one mismatch was allowed.

### 2.7. Bulk RNA-Seq Quality Control and Pre-Processing

Bulk RNA-seq data were preprocessed as previously described in Alzubi et.al. [27] Briefly, FastQC v0.11.8 [28] was used to assess sequencing quality and adapters and low-quality base pairs were removed using CutAdapt v1.15 [29]. High-quality reads were aligned to a merged human/mouse genome using STAR v2.5.2b [30] (see Alzubi et al. for merged genome construction) with the following command line options: “--outSAMtype BAM Unsorted --outSAMorder Paired --outReadsUnmapped Fastx --quantMode TranscriptomeSAM --outFilterMultimapNmax 1. The Salmon v0.8.2” [31] “quant” algorithm was used to obtain read counts from the aligned BAM files using the “IU” library type. Read counts were loaded into R to calculate Log2 TPM (transcript per million) values used for gene signature computations (methods below) and PAM50 subtyping using the genefu v2.11.2 R package [32]. New and previously generated data were analyzed (Supplementary Table S1).

### 2.8. Gene Signatures and Clustering

A set of 13 previously published gene signatures [33,34,35,36,37,38,39,40] were scored by averaging bulk RNA-seq Log2 TPM expression values over all genes in each signature to create a gene signature profile for each PDX. Morpheus was used to cluster these gene signature PDX profiles, with hierarchical clustering using the one minus Pearson’s correlation coefficient as the distance metric and average linkage method to cluster both rows and columns. PDX gene signatures were then compared using Pearson’s correlation coefficient to generate a similarity matrix.

### 2.9. Single-Cell RNA Sequencing, Quality Control, and Preprocessing

Single-cell RNA-Seq (scRNA-Seq) was performed on four TNBC cell lines (HCC1143, HCC1187, MDA-MB-468, and SUM149) and 13 PDX samples using the Chromium single Cell Gene Expression Kit (10x Genomics) per the manufacturer’s protocol and sequenced in the VCU Genomics core. The TNBC cell line samples were aligned to the GRCh38 version of the human genome and gene expression was calculated using the 10x Genomics CellRanger v6.0 software suite of tools. Dead and poor-quality cell removal was performed using an in-house R v4.1.3 script with the Seurat v4.3.0 package [41]. Briefly, cutoffs for number of genes detected (nFeature), number of molecules detected (nCount), and percent mitochondrial expression (percent.mt) were calculated for each sample individually using 3 median absolute deviations (MADs) above the median value for that sample (the median for the percent.mt attribute was calculated using only cells with ≤50% mitochondrial expression). Cells were deemed poor quality if their nFeature or nCount value was greater than 3 MADs from the median in either direction, and if the percent.mt was above the 3 MAD cutoff, or a hard cutoff of 25%, whichever was lower. PDX samples followed a similar pipeline, however, they were first aligned to the 10x Genomics merged human/mouse genome (human genome version GRCh38 and mouse genome version mm10) to identify and remove mouse cells. Once mouse cells were removed the remaining human cells were re-aligned to the GRCh38 human genome, followed by the dead and poor-quality cell filtering described above. All PDX samples were then normalized using log normalization and merged using Seurat’s merge() function. A principal component analysis (PCA) was performed using a 100-gene PI3K activity signature from Gatza et al. [33], instead of the default most variable features in the Seurat pipeline. This modified PCA matrix was utilized for the generation of UMAP and tSNE visualizations, as well as Seurat’s graph-based clustering. An in-house script was then used to export the Seurat object to a 10x Genomics formatted file, and the CellRanger “reanalyze” function was run to reformat the data so that it could be imported into the 10x Loupe Cell Browser v6. Data analysis scripts are available on GitHub at https://github.com/AmyOlex/Boyd_SynCompounds_PI3K (accessed on 31 January 2023). Note that for this study the raw data were re-processed with a newer version of the 10x CellRanger software than previously reported.

### 2.10. Western Blot

Lysates were made using homogenized tumors in RIPA with protease and phosphatase inhibitors, sonicated, and cold centrifuged at 4 °C for 20 min. Lysates were quantified using Bradford reagent using spectrophotometer (ThermoScientific BioMate 160) to quantify absorption. Electrophoresis samples were made from those lysates, Laemmli buffer, and beta-mercaptoethanol, heated to 95 °C. Electrophoresis was run using (BIO-RAD, Hercules, CA, USA, mini protein TGX 4–15% gradient) in using (BIO-RAD PowerPac Basic). Gels were transferred using semi-dry blotter (BIO-RAD Tran-Blot Turbo) onto methanol-activated nitrocellulose membranes using WypAll sheets soaked in transfer buffer. Cell Signaling anti- Vinculin, RPS6 p-RPS6 rabbit primaries were used. LI-COR 680 florescent anti-rabbit donkey were used as secondary antibodies, which were detected using (LI-COR, Lincoln, Nebraska, Odyssey FC) using ImageStudio. Drying the membrane and reimaging were utilized to reduce background. P-RPS6 levels were used as a known metric for PI3K pathway inhibition [42].

### 2.11. Ray Biotech Array C55

Ray Biotech’s C55 arrays were incubated with lysates that were produced from MDA453 and UCD52 cells, which had been treated either with DMSO control or 5 μM BYL-719, which were previously sonicated and cold centrifuged. The membranes were imaged using ImageStudio. Relative development was quantified using ImageStudioLite, each probe was normalized relative to background, those values were normalized relative to positive control probes normalized to their own background.

### 2.12. Immunohistochemistry (IHC)

Using the DAKO envision system HRP kit for rabbit primaries, formalin-fixed, paraffin-embedded tissues were cut and sections placed on slides. These sections were melted at 60 °C and rehydrated using stepwise xylene to ethanol to water baths. Antigen retrieval was performed using 9pH EDTA, TRIS Antigen retrieval buffer in a decloaking chamber (DakoCytomation, Glostrup, Denmark, Pascal). Slides were washed in TBST. A solution of 0.3% hydrogen peroxide was used as a peroxidase block. Then, slides were washed in TBST. Anti-PTEN, Anti-RPS6, and Anti-p-RPS6 rabbit primary Cell Signaling antibodies were used for overnight incubation at 4 °C. Then they were washed again in TBST. HRP-conjugated anti-rabbit secondaries were used to incubate the sections, then they were washed again in TBST before DAB incubation. Finally, they were washed in TBST one more time before hematoxylin counterstaining for 1 min, which was rinsed repeatedly with tap water. Dehydrating was performed using stepwise water to ethanol to xylene baths. Permount was used as mounting media. Slides were imaged using ZenBlue software.

### 2.13. Upstream Regulator Pathway Analysis

Normalized values from protein array data were uploaded into Ingenuity Pathway Analysis (IPA) and analyzed to produce Z scores that denote relative activity of known clusters of molecules contributing to pathway activity. IPA was also utilized to predict upstream regulators, which are molecules that it determined were likely to cause the state of uploaded expression values.

## 3. Results

### 3.1. Mutation Profiling Revealed PIK3CA Aberrations Are Common

**Figure 1:**
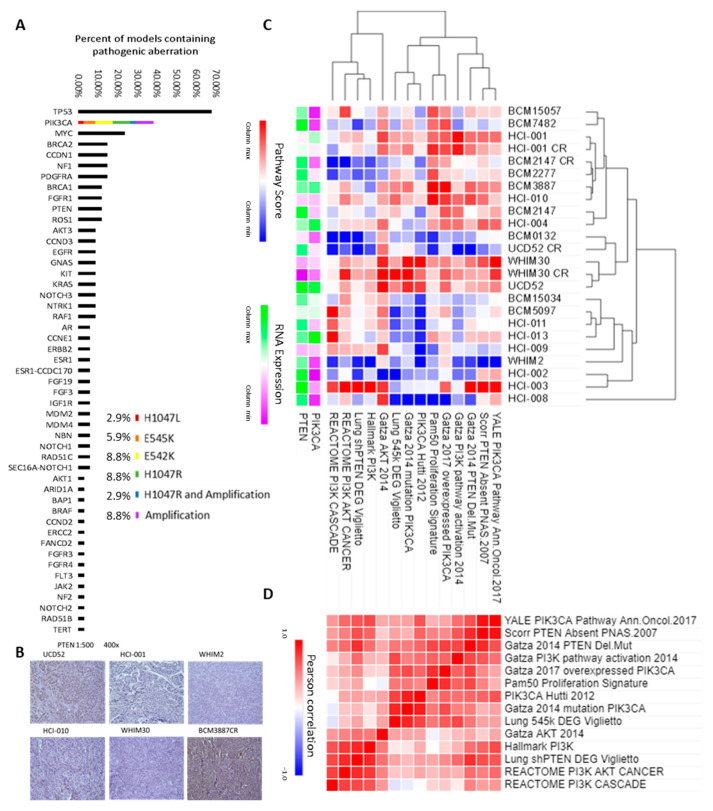
High PIK3CA gene aberration and pathway enrichment in many PIK3CA^wt^ PDX models. (**A**) Percent oncogenic aberrations. (**B**) IHC for PTEN for 6 basal-like breast cancers. (**C**) Hierarchical clustering of PDX models by previously published PIK3CA-related gene signatures and (**D**) Similarity matrix of those signatures.

To assess clinically actionable targets of these models, NGS was utilized to characterize pathogenic genetic profiles of 14 cell lines and 20 PDX samples (Table 1), which revealed pathogenic PIK3CA aberrations in 37% of models tested; 5 cell lines and 7 PDXs. PIK3CA was the second most commonly identified pathogenic driver among these models after TP53 confirmed previous observations [43]. The most common PIK3CA aberrations found in patient tumors were identified in this cohort; this included amplification through copy number gain and two of the activating mutations, E542K and H1047R (Figure 1A).

### 3.2. Binary Single Gene Status Is Not Enough to Assess PI3K Pathway Activity

Human-specific bulk RNA sequencing data of 82 samples (45 previously unpublished) from 21 PDXs were used to stratify PDX models based on their proliferation rate (11-gene proliferation signature) [44] and PI3K pathway activity defined through 13 previously published PI3K gene signatures [33,34,35,36,37,38,39,40]. Most basal-like PDXs had low PTEN expression when assessed by IHC, but BCM3887CR (a model that has become carboplatin resistant, CR) was an example of high PTEN expression (Figure 1B). Figure 1C shows the hierarchical clustering of these signatures and PIK3CA and PTEN as individual gene values overlayed for reference. UCD52 overexpresses PIK3CA and scored highly on the signatures despite high PTEN expression, which usually inhibits PI3K activity. The reason UCD52 scored highly may have been that the PTEN it expresses has a deactivating mutation (Table 1). WHIM30 and HCI-010 scored highly despite modest PIK3CA single gene expression and no pathogenic mutation, but these PDXs are PTEN deficient. WHIM2 is an example of a basal-like breast cancer that is not PI3K overactive, having moderate PTEN expression and low PIK3CA expression and scoring relatively lowly on PI3K activity signatures. WHIM2 exhibiting relatively low PI3K gene expression levels was similarly observed in luminal models (BCM15034, BCM5097, HCI-011, HCI-013, HCI-009), clustering more closely to them than to other basal-like models. Two of the ER+, HCI-011 and HCI-013, scored relatively lowly on most of these gene signatures, despite containing an activating mutation of PIK3CA. UCD52 appears to have reduced PI3K activity after treatment with carboplatin (UCD52CR), but this may be because of a reduction in average RNA expression (from 2.17 to 2.01 log2 TPM), whereas HCI-001 and WHIM30 did not have as large of a change (from 2.15 to 2.14 and from 2.16 to 2.20 log2 TPM, respectively). An all-by-all pairwise comparison of the PDX gene signature enrichment scores using Pearson’s correlation coefficient (Figure 1D) revealed these signatures were, overall, highly similar to one another. Only 4 out of 91 signature pairs obtained a negative correlation, with the lowest correlation being −0.12 (REACTOME PI3K CASCADE to Lung 545k DEG Viglietto) and the highest correlation being 0.91 (Scorr PTEN Absent PNAS.2007 to YALE PIK3CA Pathway Ann.Oncol.2017) on a scale of from −1 to 1.

### 3.3. Single-Cell RNA-Seq Identifies Cell Subpopulations with High PI3K Activity

**Figure 2:**
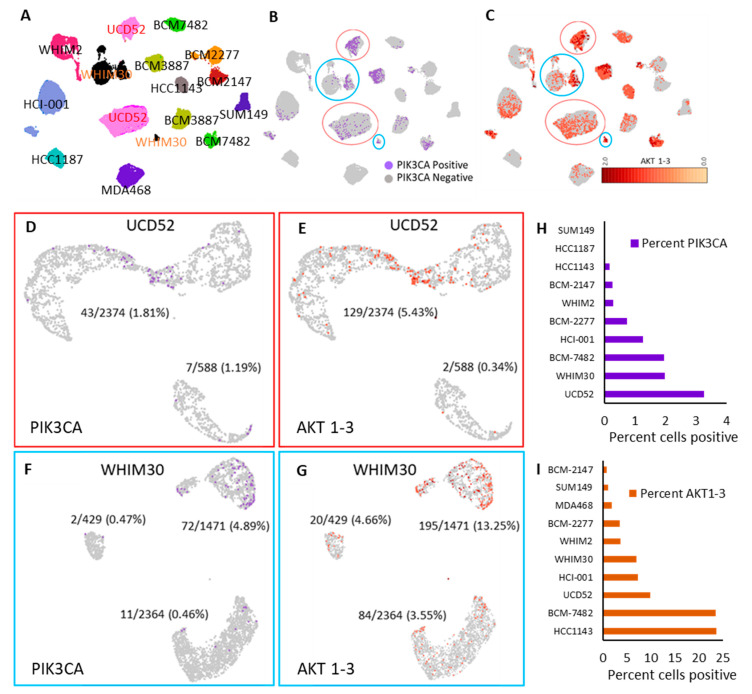
Subpopulations less reliant on the PIK3CA pathway were identified among 2 basal-like PDX models. (**A**) UMAP using the Gatza et al. The 2017 PIK3CA signature of scRNA sequencing data of basal-like models. Cells positive for (**B**) PIK3CA expression marked in purple and (**C**) by the level of AKT1-3 average expression. Unsupervised reclustering of (**D**) PIK3CA positive cells in UCD52 and (**E**) AKT 1-3 expression. Unsupervised reclustering (**F**) WHIM30 PIK3CA positive and (**G**) AKT1-3 expression. Percent positive (>0) (**H**) PIK3CA and (**I**) AKT1-3 cells per model.

Because PI3K inhibitors have not been found to be effective as single agents, we sought to determine if subpopulations of cells existed within PDXs that would be more or less therapeutically targetable for inhibitors of PI3K. Single-cell RNA-seq data containing 37,851 total cells from 18 samples (6 published previously) across 12 models (4 cell lines and 8 PDX) were utilized to compare cells and subpopulations. A 100-gene PI3K activity signature from Gatza et al. [33] was used to map these models, some of which, such as UCD52 and WHIM30, formed distinct subpopulations. (Figure 2A) These populations contained different proportions of the PIK3CA single gene (Figure 2B) and AKT1, AKT2, and AKT3 average expression (Figure 2C). Individual re-cluster analyses of UCD52 or WHIM30 cells identified two or three distinct subpopulations within each PDX (Figure 2D–G). Each model in the cluster was assessed for PIK3CA and AKT1-3 percent cell positivity (>0) (Figure 2H,I).

### 3.4. Alpha-Specific PIK3CA Inhibitors Were More Effective on PIK3CA Aberrant Cells

**Figure 3:**
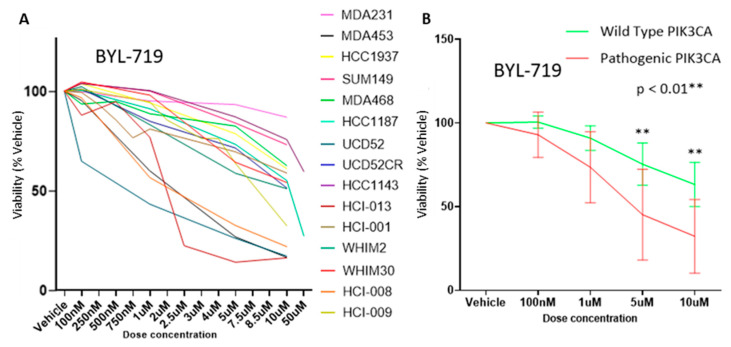
Pathogenic PIK3CA-containing models were more responsive to BYL-719. (**A**) Dose to viability by model in vitro. (**B**) Dose to viability by, grouped by PIK3CA oncogenic status.

To determine if the PIK3CA aberrations found within the models tested were targetable and resulted in the loss of cell viability, two clinically tested PI3K inhibitors (PI3Ki) were utilized; BYL-719 and GDC-0032 (marketed as taselisib). HCI-013, an ER+ PIK3CA^H1047R^ mutant PDX, was the most responsive to both drugs at higher doses (Figure 3A and Supplemental Figure S1A). When grouped by pathogenic PIK3CA status, those with mutations were significantly (*p* > 0.01) more responsive on average to the PI3Ki in vitro at 5 μM and 10 μM using two-way ANOVA with Šidák’s correction for multiple comparisons (Figure 3B and Supplemental Figure S1B).

### 3.5. Inhibition of PIK3CA Yielded Related Predicted Upstream Regulators in Pathway Analysis

**Figure 4:**
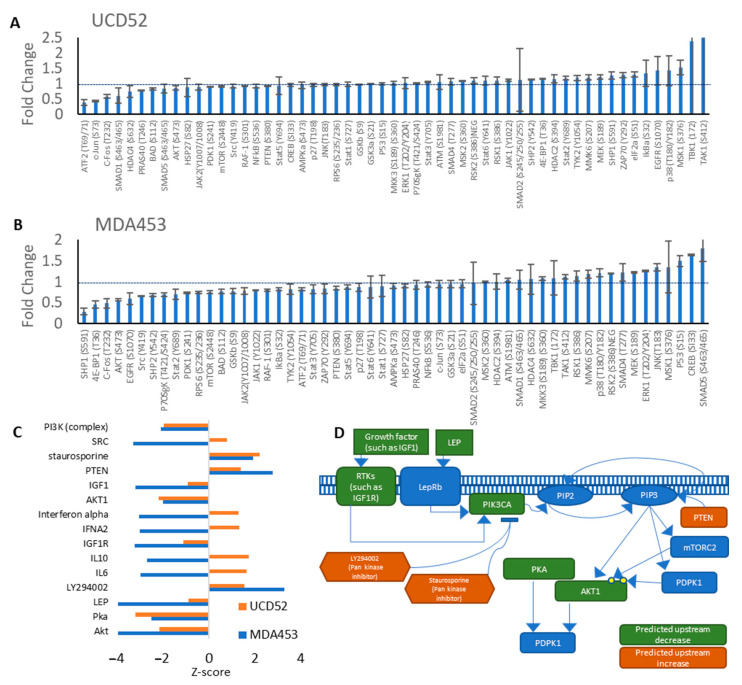
Confirmation of BYL-719’s PIK3CA inhibitory effects. Fold changes of phosphoprotein levels for (**A**) UCD52 and (**B**) MDA-MB-453 from phosphoprotein antibody arrays. (**C**) Highest z-score predicted upstream molecules in Ingenuity Pathway Analysis. (**D**) Model of how the highest predicted upstream regulators would share downstream effects with PI3K pathway suppression.

We next sought to contrast the downstream effects of PI3K inhibition on TNBC cells from a PDX as compared to a cell line often utilized for PI3K inhibition studies, MDA-MB-453. Protein lysates from two basal-like models containing pathogenic PIK3CA aberrations, UCD52 and MDA-MB-453, were prepared from in vitro cultures, which were treated with BYL-719 or vehicle and applied to antibody arrays for orpheus-proteins relating to the PI3K/mTOR/AKT pathway (Supplemental Figure S2). Phospho-protein levels were quantified and analyzed using IPA, and fold changes (Figure 4A,B) were used to predict upstream regulators. The effects of BYL-719 were predicted by IPA to be seven upstream regulators to be downregulated and three upstream regulators to be upregulated, including PTEN, of the top 15 z-scores (Figure 4C,D). PTEN downstream effects are the same as BYL-719, to reduce PIP3 levels in the plasma membrane, as PI3K activity is to convert PIP2 into PIP3, so a reduction in PIK3CA activity from BYL-719 would be predicted to have a similar or potentially identical downstream effect as an increase in PTEN activity.

### 3.6. Identification of More Effective Single Compounds Relative to PIK3CA Mutational Status

**Figure 5:**
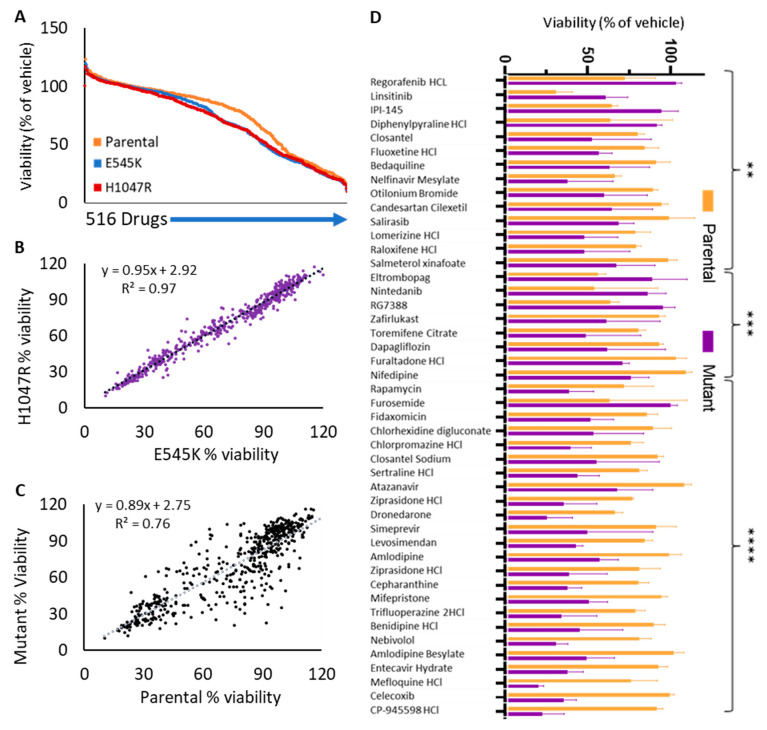
High throughput viability screening of 516 drugs on MCF10A^WT^, MCF10A^E545K^, and MCF10A^H1047R^ revealed drugs that were more differentially effective relative to PIK3CA mutation status. (**A**) Drugs on each variant are ranked by model by viability. (**B**) Each drug’s effect on the viability of both mutant-containing models was compared. (**C**) The average of both mutant-containing models’ viability when treated with each drug compared to parental viability for that drug. (**D**) Significant (*p* < 0.01) drugs when comparing MCF10A^WT^ viability to that of the average MCF10A mutant viability. (** denotes *p* < 0.01, *** denotes *p* < 0.001, **** denotes *p* < 0.0001).

Since the sensitivity or resistance to drugs that were not intended for targeted treatment can arise from a mutation in another gene, it was important to test the effect of PIK3CA activating mutations in controlled models. MCF10A wild type, E545K, and H1047R PIK3CA variant containing cell lines, when screened, responded to a similar number of drugs at 40% viability or lower (Figure 5A). The two mutant variants responded similarly (R = 0.97) to each drug as one another (Figure 5B), but when the average value of each of those was compared to the wild type containing cell line, viability was more dissimilar (R = 0.76) (Figure 5C); generally, each oncogenic mutant version caused cells to react the same to most drugs, but having one of these mutations changes the response of the cell to far more drugs relative to wild type containing cells. Different viability in parental versus mutation-containing cells was observed in 45 drugs, significant at 0.01 or lower *p*-value using a two-way ANOVA correcting for multiple comparisons using Tukey’s post-hoc test (Figure 5D). No drugs were significant with the same statistical comparison when comparing the effects of those drugs relative to mutation.

### 3.7. Identification of Synergistic Compounds with BYL-719

**Figure 6:**
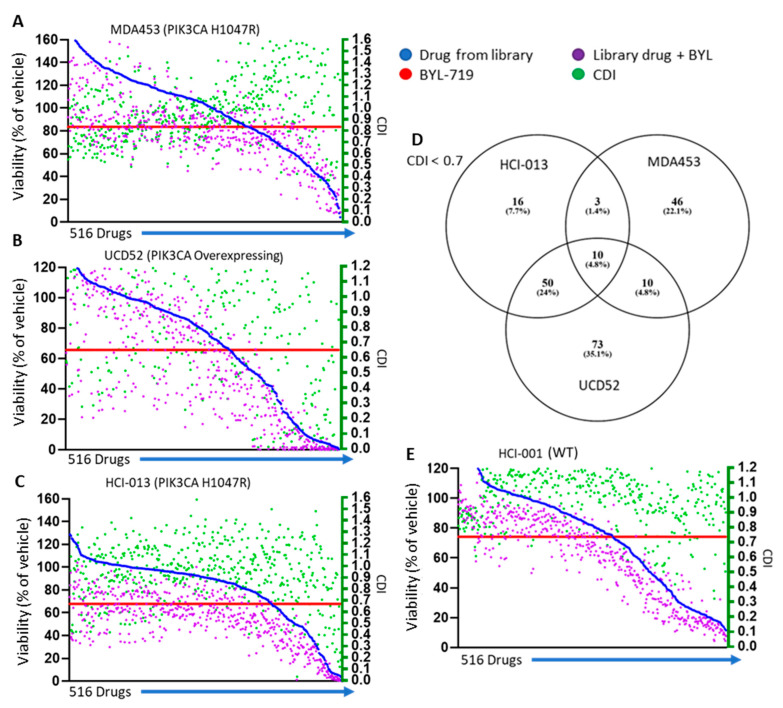
High throughput synergism screening revealed drug combinations of interest. A library of 516 drugs with and without BYL-719 with the viability of each drug (left *x*-axis) and CDI (right *x*-axis) for each drug combination in (**A**–**C**) PIK3CA pathogenic models and (**D**) Venn diagrams of drugs under 0.7 CDI for oncogenic PIK3CA containing models. (**E**) Viability of single and combinatorial agents and CDI in the PIK3CA^WT^ containing HCI-001.

Because BYL-719 as a single agent only had modest effects in vivo (Supplemental Figure S3A) while still inhibiting PI3K activity (Supplemental Figure S3B), it was of interest to discover agents that potentiated BYL-719’s effect on these models. High-throughput screening (HTS) with 516 single drugs alone and those agents with BYL-719 was performed on MDA453, UCD52, and HCI-013 (Figure 6A–C). Of drugs that were found to be synergistic using the coefficient of drug interaction (CDI), there were 20 for the two basal-like PIK3CA oncogenic aberration containing model, and half of those were also found to be synergistic with BYL-719 in HCI-013, an ER+ model which also has oncogenic PIK3CA. In total, 20 drugs were identified as synergistic in both basal-like PIK3CA aberrant-containing models, half of which were also synergistic in the ER+ PIK3CA^H1047R^ (Figure 6D). Some drugs were highly effective as single agents, such as YM-155 and Digoxin, so significant synergy was not observed in combination with BYL-719. The PIK3CA^WT^ basal-like PDX HCI-001 had fewer drugs with which BYL-719 was synergistic, and none of those found in the aberrant models were found to be synergistic in HCI-001 (Figure 6E).

### 3.8. CompuSyn Was Utilized to Confirm Synergistic Effects of Candidate Therapies

**Figure 7:**
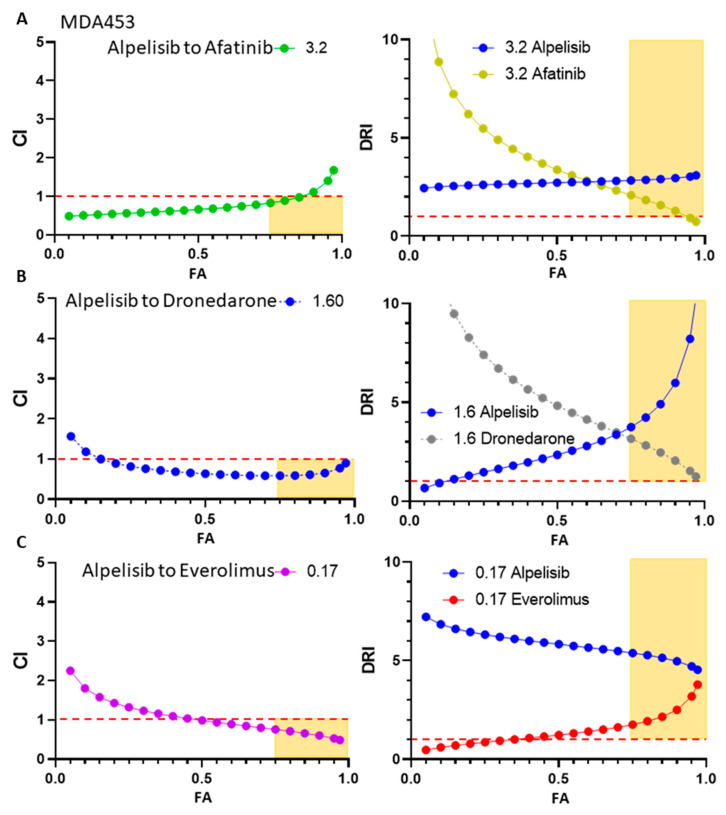
Compusyn was utilized to confirm synergism with higher rigor and to determine effective dose ratios in vitro on MDA453 cells. (**A**) Projected combination indexes per fractions affected for BYL-719 and afatinib and projected dose-reduction indexes per fractions affected for each drug at the dose ratio, BYL-719 to afatinib, listed. The same is shown for the combinations of BYL-719 with (**B**) dronedarone and (**C**) everolimus.

To investigate these drugs at a higher level of rigor, afatinib, dronedarone, and everolimus, three of the drugs which were discovered in HTS, were assessed with CompuSyn using 5 dose ratios per drug and 5 or more dose escalations per ratio in MDA453 (Supplemental Figure S4). Each of these combinations was predicted to have at least one dose ratio that was synergistic below one combination index (CI) and at a high fraction affected (FA), denoting that each combination had at least one dose ratio that performed better than the expected effects of both drugs alone combined. Each combination was predicted to have dose reduction index (DRI) greater than one at high FA with at least one dose ratio (Figure 7A–C), denoting that each combination could have one or the other of the drug’s concentrations reduced and be predicted to see the same effects. Highlighted in yellow is the range of interest, FA of 0.75 or higher, and CI below one or DRI above one.

### 3.9. In Vitro Synergism Testing of Select Dose Ratios of Synergistic BYL-719 Combinations

**Figure 8:**
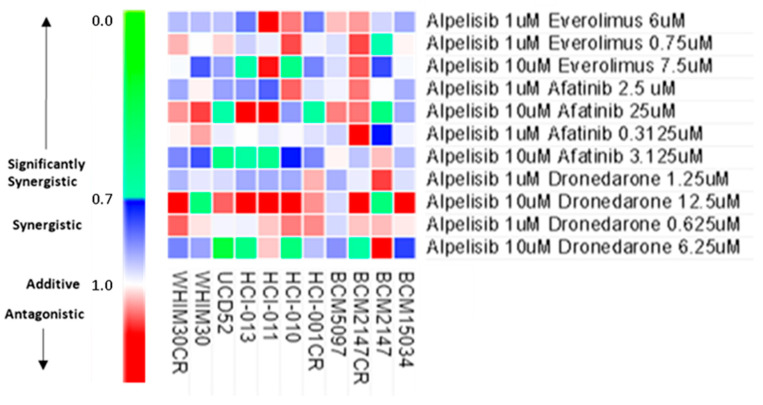
In vitro synergism screening revealed synergistic dose ratios across multiple PDX models. CDI for each drug combination at 4 dose ratios each, green denotes significant synergism, blue denotes synergism, and red denotes antagonistic effects, while white is additive.

Those three drug combinations were then tested at two dose ratios each, each at a lower and higher escalation of doses, for four conditions total per drug combination. In total, 11 models were tested in vitro. Two dose ratios showed synergistic to significantly synergistic response in all models tested, the BYL-719 10 μM with afatinib 3.125 μM combination and the same dose of BYL-719 with 6.25 μM dronedarone. No dose ratio with everolimus showed synergism across each model, but the 10 μM BYL-719 with 7.5 μM everolimus had synergistic to significantly synergistic proliferation reduction in HCI-013, UCD52, WHIM30, and WHIM30CR. The only models that these dose ratios that were not synergistic were the ER+ HCI-011, which does contain pathogenic PIK3CA, and the PIK3CA^WT^, PTEN^WT^ basal-like PDX, BCM2147 (Figure 8).

### 3.10. In Vivo BYL-719 Synergism Trial on PI3K Overactive Basal-like PDXs

**Figure 9:**
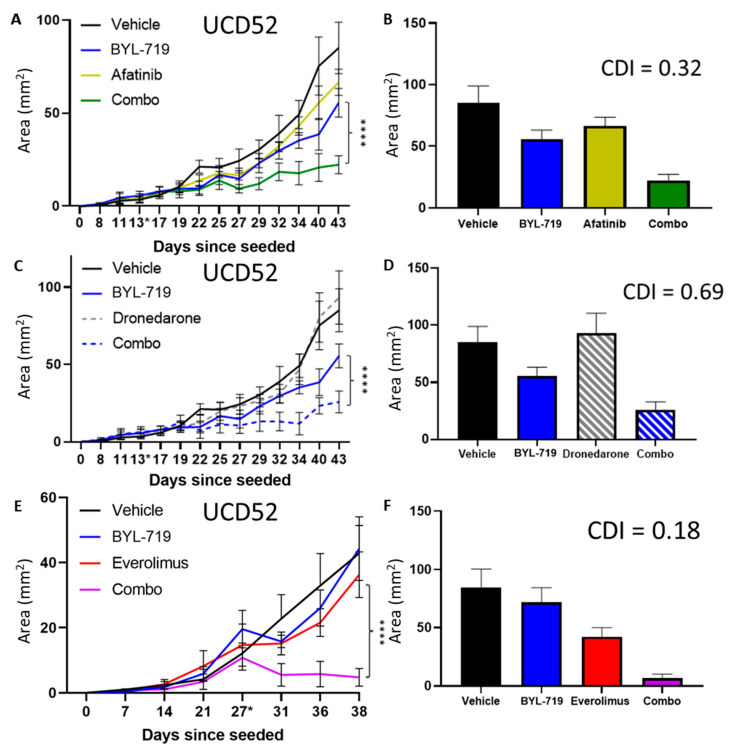
The combinations of everolimus, afatinib, or dronedarone with BYL-719 were significant and significantly synergistic in UCD52. BYL-719 tested in vivo in UCD52, comparing to alternative single agents, (**A**,**B**) afatinib, (**C**,**D**) dronedarone, and (**E**,**F**) everolimus and the combination of each with BYL-719. **** denotes *p* < 0.0001 significance, ANOVA after Tukey correction for multiple comparisons, comparing final tumor area. * on the days since seeded axis denotes the day that the treatment was started.

Finally, the drugs needed to be tested in living systems. BYL-719 alone, afatinib alone, the combination, and vehicle control were each administered to mice bearing tumors of the PIK3CA overactive UCD52 basal-like PDX. The combinatorial group showed significant (*p* < 0.0001) and significantly synergistic activity when comparing the final tumor area (CDI = 0.32) (Figure 9A,B). The combination of dronedarone and BYL-719 was significantly more effective than either agent alone (*p* < 0.0001) (Figure 9C). Everolimus with BYL-719 also showed significant efficacy compared to its single-agent components (*p* < 0.0001) (Figure 9E). Each of these combinations yielded significant synergism (CDI = 0.69 and 0.18, respectively) (Figure 9D,F). PI3K pathway activity was assessed using IHC and Western blot of p-RPS6 relative to total RPS6. Each group treated with BYL-719 alone does not show the same reduction in p-RPS6 that shorter in vivo trials show, which also occurred after BYL-719 single-agent resistance began. The afatinib + BYL-719 group had reduced total RPS6 (Supplemental Figure S5), as did each group treated with dronedarone on IHC (Supplemental Figure S6). Treatment with everolimus in UCD52 reduced p-RPS6 even at longer time points. (Supplemental Figure S7).

**Figure 10:**
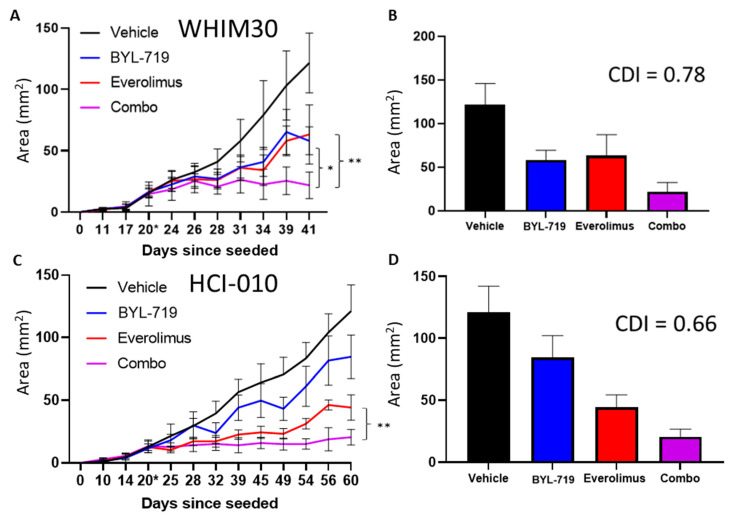
The combination of everolimus with BYL-719 performed better than the expected additive effects of both drugs in the PTEN-deficient WHIM30 and HCI-010. BYL-719 tested in vivo in WHIM30, compared to alternative single agents everolimus and the combination with BYL-719 in (**A**,**B**) WHIM30 and (**C**,**D**) HCI-010. * denotes *p* < 0.05, ** denotes *p* < 0.001 significance, ANOVA after Tukey correction for multiple comparisons, comparing final tumor area. * on the days since seeded axis denotes the day that the treatment was started.

These three combinations were tested in the same way on mice with xenografts of WHIM30, a PTEN deficient PIK3CA wild type basal-like PDX, with the everolimus and BYL-719 combination showing a reduction in tumor growth for each treatment group relative to the vehicle and synergistic effects (*p* < 0.05 and CDI 0.77) (Figure 10A,B) and everolimus treatment also reduced p-RPS6 in HCI-010 (Supplemental Figure S8). The combinations of dronedarone and afatinib with BYL-719 were tested, and while showing a trend, the effectiveness of BYL-719 alone drove the phenotype statistically (Supplemental Figure S9). The BYL-719 and everolimus combination was tested in HCI-010, another PTEN deficient PIK3CA wild type basal-like PDX, yielding significant difference (*p* < 0.01) and significantly synergistic effects (0.66) (Figure 10C,D). Once again, each everolimus-treated group had a reduction of p-RPS6 (Supplemental Figure S10).

## 4. Discussion

BYL-719 is currently approved for some cases of PIK3CA mutated ER+ breast cancer, though such as with HCI-011, targeting PIK3CA in ER+ disease, even with pathogenic PIK3CA, does not always yield an effective treatment.

In clinical trials not related to breast cancer, the most common side effects of dronedarone were nausea at about 5% and diarrhea at about 10%, a very manageable adverse event profile, at least as a single agent [45]. Dronedarone, a multi-ion channel inhibitor, and afatinib, an EGFR inhibitor, have been studied in breast cancer previously [46,47] but not in combination with BYL-719. Afatinib has been approved as a single agent in some lung cancers [48], but despite clinical trials so far in the breast cancer setting, it has not been shown to be effective as a single agent in breast cancer [49]. These combinations did show synergistic effects in the PI3K overactive basal-like breast cancers tested. The combination of BYL-719 plus everolimus, an mTOR inhibitor, has been studied in PIK3CA mutant breast cancer cell lines previously [50] and was reconfirmed through this study. Toxicity has been tested in a phase 1b clinical trial for HR+ HER2- breast cancer to test the safety of the drugs, resulting in a manageable safety profile with no observed interactions between the two drugs [51], which is a promising outcome since both drugs have serious, but manageable side effects alone. The efficacy of the drugs was not able to be assessed in that trial because of the sample size. The results of previous research of the BYL-719 and everolimus combination point towards utilizing it on PIK3CA mutant cancers only, but they showed to be synergistic and effective in the PTEN deficient PIK3CA WT PDXs WHIM30 and HCI-010, suggesting that the precision medicine potential of BYL-719 plus everolimus should be considered in the treatment for PTEN lacking basal-like cancers, which are three or more times or more common than PIK3CA mutation containing basal-like breast cancer.

## 5. Conclusions

Further research is merited, but based on these results, clinical trials could be considered using BYL-719 in combination with everolimus for basal-like tumors with PI3K pathway overactivity, either through PTEN loss or pathogenic PIK3CA and the combinations of either dronedarone or afatinib with BYL-719 should be considered for testing for patients with basal-like breast cancer with pathogenic PIK3CA.

## Figures and Tables

**Table 1:** Oncomine V3 revealed clinically actionable pathogenic calls. The 34 BRCR models probed with Oncomine sequencing of 229 targeted oncogenic aberrations, returning the status of known mutations, DNA CNVs, and RNA fusion drivers. Models with genes with * were sequenced twice, those marked appeared both times. Pathogenic aberrations in PIK3CA are highlighted in red and in PTEN are highlighted in orange. “CR” denotes treatment with carboplatin.

Name	Model	Oncomine Mutation	Oncomine Amplification	Oncomine Fusion
HCC1143	Cell line	TP53 (c.743G>A)	CCND1, FGF19, FGF3, KIT, MDM2, PDGFRA	
HCC1187	Cell line	BAP1 (c.781C>T), TP53 (c.322_324delGGT)	TP53, CCND3	
MCF7	Cell line	PIK3CA (c.1633G>A)	GNAS, RAD51C	ESR1-CCDC170
MDA231	Cell line	BRAF (c.1391G>T), KRAS (c.38G>A), NF1 (c.1398_1399insC), NF2 (c.691G>T), NOTCH3 (c.1102A>T), TERT (c.-124C>T), TP53 (c.839G>A)		
MDA453	Cell line	FGFR4 (c.1100A>G), PIK3CA (c.3140A>G), PTEN (c.919G>A)	CCND1, ERBB2, MDM4	
MDA468	Cell line	PTEN (c.253+1G>T), TP53 (c.818G>A)	CCNE1, EGFR	
SUM149	Cell line	BRCA1 (c.2169delT), NF1(c.4195C>T), TP53 (c.711G>A)		
T47D	Cell line	ARID1A (c.2830C>T), PIK3CA (c.3140A>G), TP53 (c.580C>T)	PIK3CA	
UCD115	Cell line	TP53 (c.892_911dupGAGCTGCCCCCAGGGAGCAC)	ERCC2, MYC, ROS1	
UCD12	Cell line	BRCA2 (c.4943delC), PIK3CA (c.3140A>T)	FGFR1, RAD51C	
UCD178	Cell line	NF1 (c.6322_6323insG)	AKT3, MYC, NTRK1	SEC16A-NOTCH1
UCD4	Cell line	BRCA2 (c.1755_1759delGAAAA), ESR1 (c.1613A>G), RAD51B (c.246C>G)	FANCD2, FGFR3, RAF1	
UCD46	Cell line	TP53 (c.281C>G)	CCND2, PIK3CA	
UCD65	Cell line	NF1 (c.2372dupT), NOTCH2 (c.6403_6404delCT)	CCND1, FGF19, FGF3, FGFR1, GNAS	
BCM0132	PDX	TP53* (c.524G>A)	MYC, NTRK1	
BCM15034	PDX	NOTCH3 (c.1487_1488insT)	ERBB2	
BCM15057	PDX	NOTCH1 (c.5402C>G), TP53 (c.438G>A), PIK3CA* (c.1624G>A)	CCND1, FGFR1, MDM2	ESR1-CCDC170
BCM2147	PDX	PDGFRA (c.2471dupT)	MYC	
BCM2277	PDX	NOTCH3 (c.3060dupC; n.-1413dupC), KRAS (c.182A>G)		
BCM3887	PDX	TP53* (c.438G>A), BRCA1 (c.5406+5G>T)		
BCM5097	PDX	BRCA2 (c.3545_3546delTT), NOTCH1* (c.3835C>T)		
BCM7482	PDX	BRCA1* (c.5177_5180delGAAA; c.650_653delGAAA), TP53* (c.586C>T) NF1 (c.925G>A)	FLT3, RAF1, IGF1R	
BCM7821	PDX	TP53* (c.330_333delTCTG)	CCNE1, MYC, PDGFRA, IGF1R	
HCI-001	PDX	BRCA2* (c.3847_3848delGT), TP53* (c.783delT), AR (c.1127C>T)	AKT3, CCND3*, MYC*, NBN, ROS1*,	
HCI-001CR	PDX	BRCA2 (c.3847_3861delGTAAGTGAAAAAAAT), TP53 (c.783delT)	CCND3, MYC, ROS1	
HCI-008	PDX	PIK3CA (c.3140A>G), TP53 (c.818G>A)	CCND1, EGFR, RAF1, ROS1	
HCI-009	PDX	PIK3CA (c.1624G>A)	AKT1	SEC16A-NOTCH1
HCI-011	PDX	PIK3CA (c.1633G>A), TP53 (c.993+1G>T)	FGFR1, GNAS	
HCI-013	PDX	ESR1 (c.1610A>C), PIK3CA (c.3140A>G)		
UCD52	PDX	TP53 (c.880G>T), PTEN (c.368A>C)	PIK3CA	
UCD52CR	PDX	TP53* (c.880G>T), PTEN* (c.368A>C)	KIT, KRAS*, MYC*, PDGFRA, PIK3CA*	
WHIM2	PDX	TP53 (c.497dupC)	EGFR, KIT, NTRK1, PDGFRA, AKT3, AR, JAK2, MDM4	
WHIM30	PDX	TP53 (c.375+2T>A)	NBN	
WHIM30CR	PDX	TP53 (c.375+2T>A)		

## Data Availability

The scRNA seq data presented in this manuscript is available online. Full Western blots are included in the supplemental figures. All other data used, including genes which comprise each gene signature, was submitted as supplemental uploads to the journal.

## Supplementary Materials

The following supporting information can be downloaded at: https://www.mdpi.com/article/10.3390/cancers15051582/s1, Supplemental Table S1. Bulk and scRNA samples used. Supplemental Figure S1. Pathogenic PIK3CA-containing models were more responsive to GDC-0032. (A) Dose to viability by the model in vitro. Dose to viability by model, grouped by PIK3CA oncogenic status. Supplemental Figure S2. Phospho-protein array membranes of (As) untreated and treated (Bs) from lysates made with UCD52 and MDA453 treated with BYL-719. Each number denotes the numbered membrane from the RayBiotech C55 phosphoprotein array. Supplemental Figure S3. Preliminary single-agent trials of BYL-719 showed some efficacy in WHIM30 and UCD52, but resistance arose. In HCI-001 and BCM2147CR6, the drug performed as well as the vehicle. Supplemental Figure S4. Full output from CompuSyn shows multiple drug ratios that were effective in MDA453 for combination index (CI) and for dose reduction index (DRI) and high fraction affected (FA). Supplemental Figure S5. Treatment did not show a reduction in p-RPS6. (A) H + E and IHC of RPS6 and p-RPS6 of each treatment group in the BYL-719 with Afatinib trial in UCD52. (B) Western blots of Vinculin loading control and RPS6 and p-RPS6. Supplemental Figure S6. Total RPS6 was reduced with dronedarone treatment in UCD52 in IHC but not in western. (A) H + E and IHC of RPS6 and p-RPS6 of each treatment group in the BYL-719 with Dronedarone trial in UCD52. (B) Western blots of Vinculin loading control and RPS6 and p-RPS6. Supplemental Figure S7. The levels of p-RPS6 were reduced with treatment of either BYL-719 or Everolimus and even further with combination therapy in UCD52. (A) H + E and IHC of RPS6 and p-RPS6 of each treatment group in the BYL-719 with Everolimus trial in UCD52. (B) Western blots of Vinculin loading control and RPS6 and p-RPS6. Supplemental Figure S8. The levels of p-RPS6 were reduced with treatment of either BYL-719 or Everolimus and even further with combination therapy in WHIM30. (A) H + E and IHC of RPS6 and p-RPS6 of each treatment group in the BYL-719 with Everolimus trial in WHIM30. (B) Western blots of Vinculin loading control and RPS6 and p-RPS6. Supplemental Figure S9. Further combination therapies showed a trend of an effect in WHIM30. The final tumor area of the combinations of (A + B) Afatinib and (C + D) Dronedarone with BYL-719 was reduced relative to either treatment alone, but the effect of the combination was not significant relative to the effect of BYL-719 alone. Supplemental Figure S10. The levels of p-RPS6 were reduced with the treatment of Everolimus in HCI-010. (A) H + E and IHC of RPS6 and p-RPS6 of each treatment group in the BYL-719 with Everolimus trial in HCI-010. (B) Western blots of Vinculin loading control and RPS6 and p-RPS6. Supplemental Figure S11. Complete Western blots of in vivo trials from tumors of the UCD52, WHIM30, and HCI-010 PDXs that were treated with vehicle, single agents, or combinations. Supplemental Figure S12. Complete Western blots of in vivo trials from tumors of the UCD52, WHIM30, and HCI-010 PDXs that were treated with vehicle, single agents, or combinations values indicate densitometry.
