# Supplementary material for: Discovering Synergistic Compounds with BYL-719 in PI3K Overactivated Basal-like PDXs
Source: Cancers (Basel). 2023 Mar 3;15(5):1582. doi: 10.3390/cancers15051582 (PMC10001201; doi:10.3390/cancers15051582)
Supplement: Supplementary file 1 [file cancers-15-01582-s001.zip › cancers-2223650-supplementary.pdf]

GSE	Samples (bulk)		GSE	Samples (sc)
GSE118942	HCI01.MGT.101020<br>HCI01.MGT.101021<br>HCI01.MGT.101022<br>HCI02.MGT.100945<br>HCI02.MGT.100946<br>HCI02.MGT.100948<br>HCI03.MGT.102031<br>HCI04.MGT.100763<br>HCI08.MGT.100879<br>HCI09.MGT.100241<br>HCI09.MGT.100508<br>HCI09.MGT.100511<br>HCI09.MGT.100512<br>HCI10.MGT.100276<br>HCI10.MGT.100277	HCI13.MGT.100822<br>PT52.MGT.100302<br>PT52.MGT.100303<br>PT52.MGT.100304<br>PT52.MGT.100775<br>PT52.MGT.100916<br>PT52.MGT.100917<br>WHIM2.MGT.100000<br>WHIM2.MGT.100027<br>WHIM2.MGT.100076<br>WHIM30.MGT.100431<br>WHIM30.MGT.175278A<br>WHIM30.MGT.181040A<br>WHIM30.MGT.182696A<br>WHIM30.MGT.NSG21A	GSE174391	HCC1143<br>HCC1187<br>MDA468<br>SUM1493
GSE189325	WHIM2.107647<br>WHIM2.107647b	WHIM2.107668<br>WHIM2.107672	GSE189324	WHIM2_106305<br>WHIM2_106361
In process	BCM.0132.107564<br>BCM.15034.107894<br>BCM.15057.107957<br>BCM.15057.107968<br>BCM.2147.107741<br>BCM.2147.107744<br>BCM.2147.107745<br>BCM.2147CR2.107841<br>BCM.2147CR2.107842<br>BCM.2277.107573<br>BCM.3887.107696<br>BCM.5097.107568<br>BCM.5097.107931<br>HCI.001.107935<br>HCI.011.107898<br>HCI.013.107857<br>HCI.013.107939<br>UCD52CR21.107961<br>UCD52CR21.107964<br>UCD52CR21.107966<br>UCD52CR21.107967<br>WHIM30.107947	WHIM30CR6.107919<br>WHIM30CR6.107920<br>WHIM30CR7.107949<br>WHIM30CR7.107950<br>WHIM30CR7.107951<br>BCM.15034.107510<br>BCM.15057.107336<br>BCM.5097.107567<br>BCM.7482.107504<br>HCI.001.100097<br>HCI.011.107518<br>HCI.011.107522<br>HCI.013.102249<br>UCD52.100398<br>UCD52.100916<br>UCD52.107627<br>UCD52CR.107532<br>UCD52CR.107591<br>UCD52CR.107592<br>WHIM30.107661<br>WHIM30.107662<br>WHIM30CR.107502	In progress	BCM-2147_107007<br>BCM-2277_107010<br>BCM-3887_107156<br>BCM-7482_107157<br>HCI-001-105983<br>HCI-001-105984<br>UCD52<br>UCD52CR<br>UC52CR_107080-107081<br>WHIM2_105940<br>WHIM30_105954

Supplemental Table S1. Bulk and scRNA samples used.

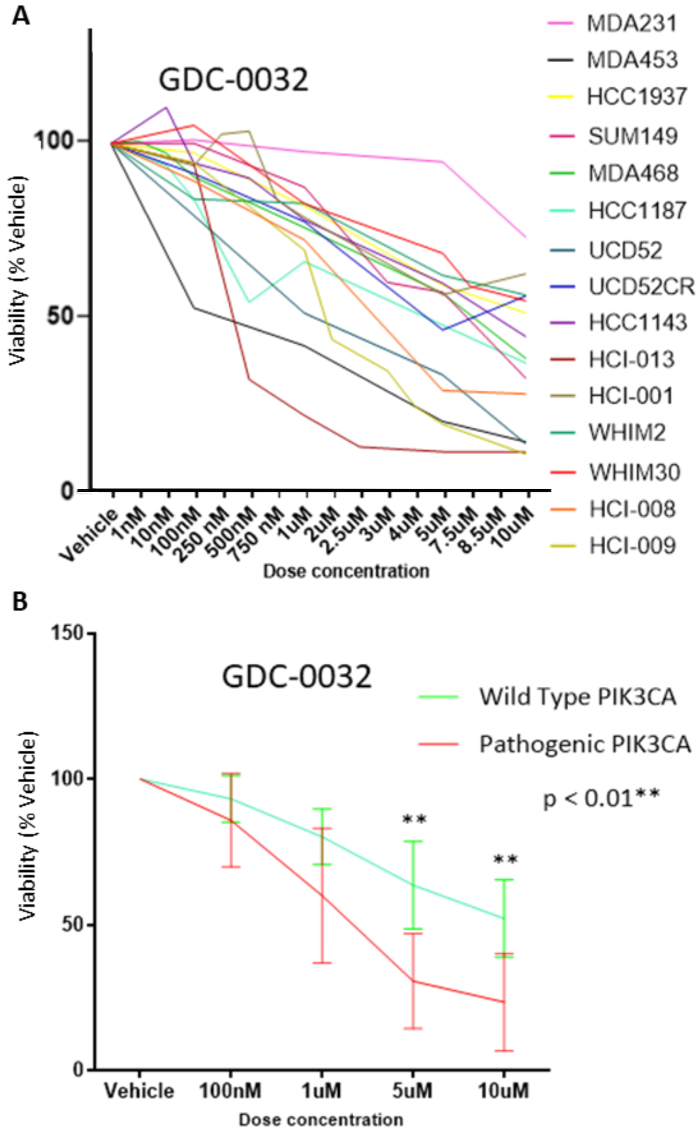

Supplemental Figure S1. Pathogenic PIK3CA containing models were more responsive to GDC- 0032. (A) Dose to viability by model *in vitro*. (B) Dose to viability by model, grouped by PIK3CA oncogenic status

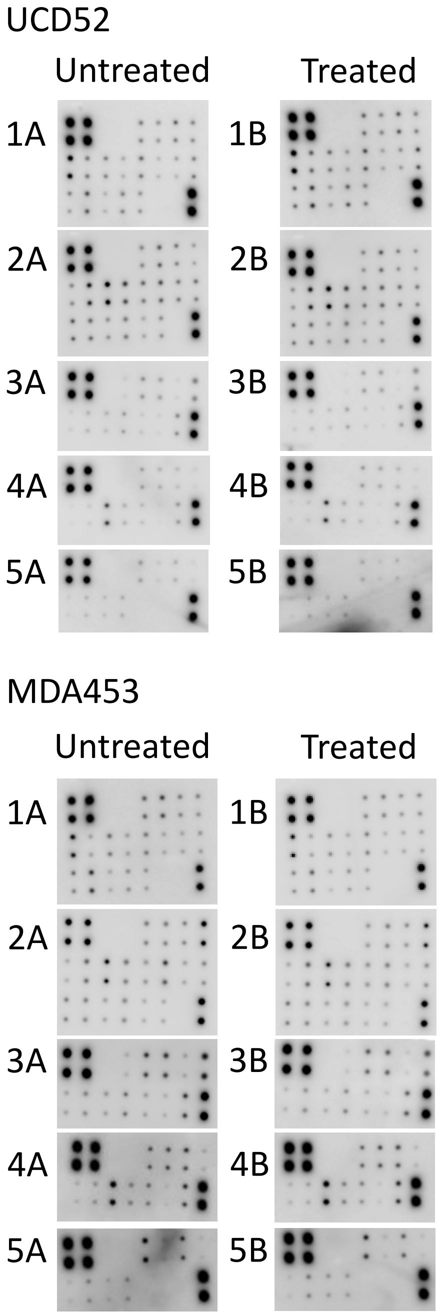

Supplemental Figure S2. Phospho-protein array membranes of (As) untreated and treated (Bs) from lysates made with UCD52 and MDA453 treated with BYL-719. Each number denotes the numbered membrane from the RayBiotech C55 phosphoprotein array.

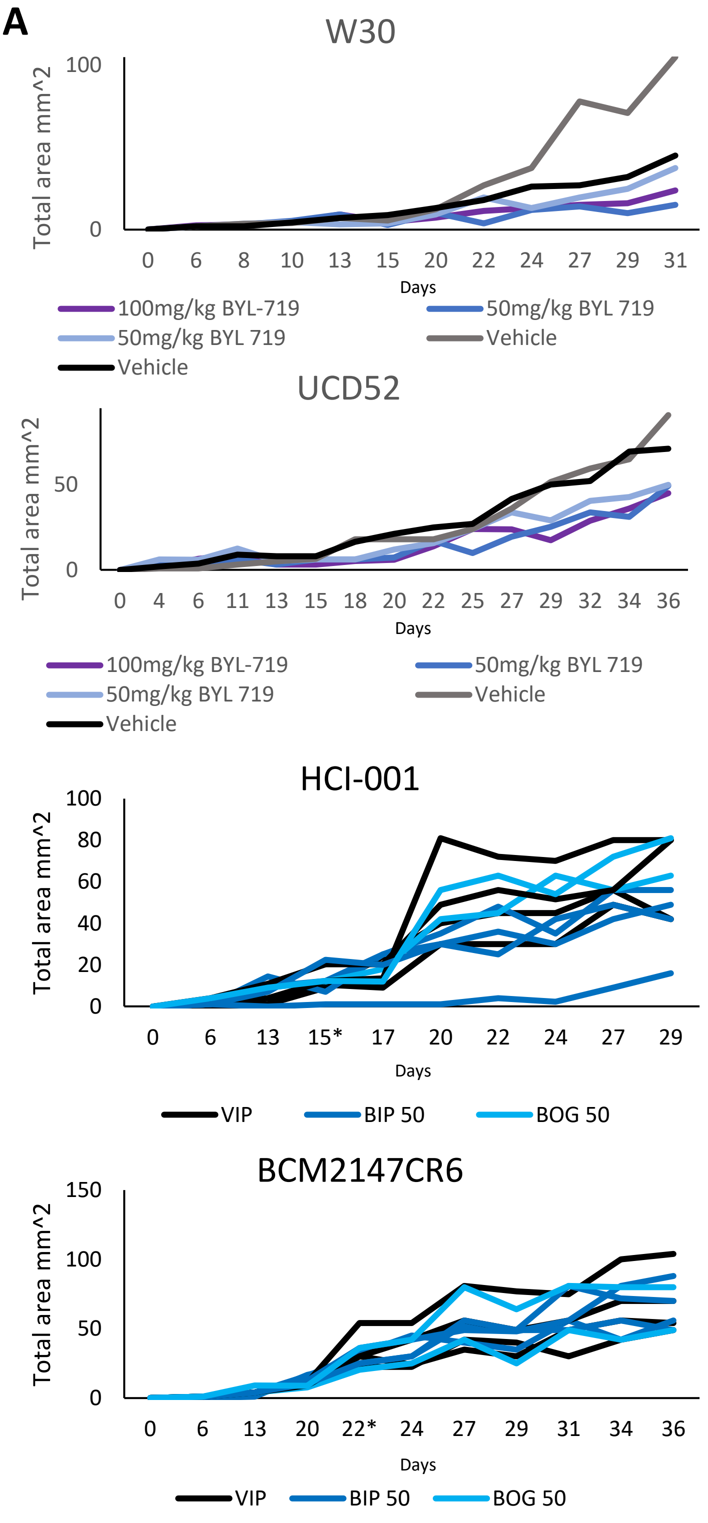

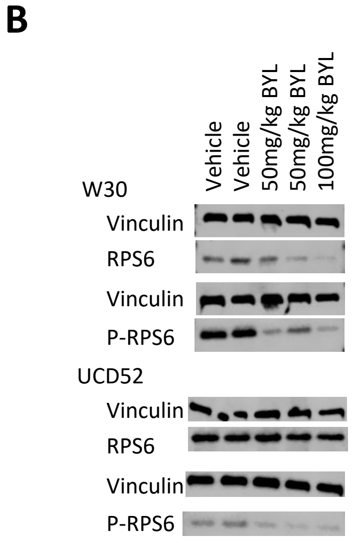

Supplemental Figure S3. Preliminary single agent trials of BYL-719 showed some efficacy in WHIM30 and UCD52, but resistance arose. In HCI-001 and BCM2147CR6, the drug preformed as well as vehicle.

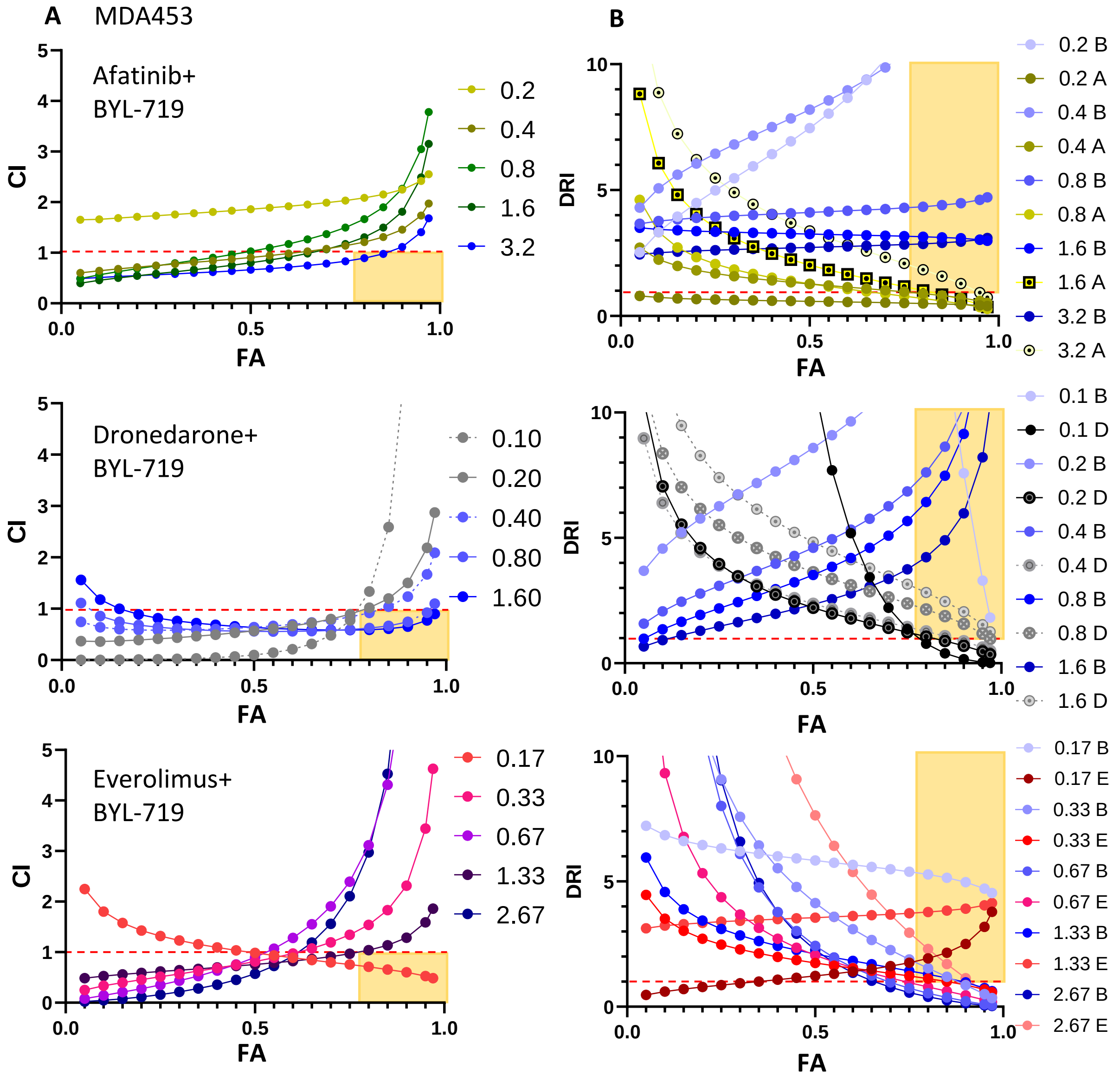

Supplemental Figure S4. Full output from CompuSyn shows multiple drug ratios which were effective in MDA453 for Combination Index (CI) and for Dose Reduction Index (DRI) and high Fraction Affacted (FA)

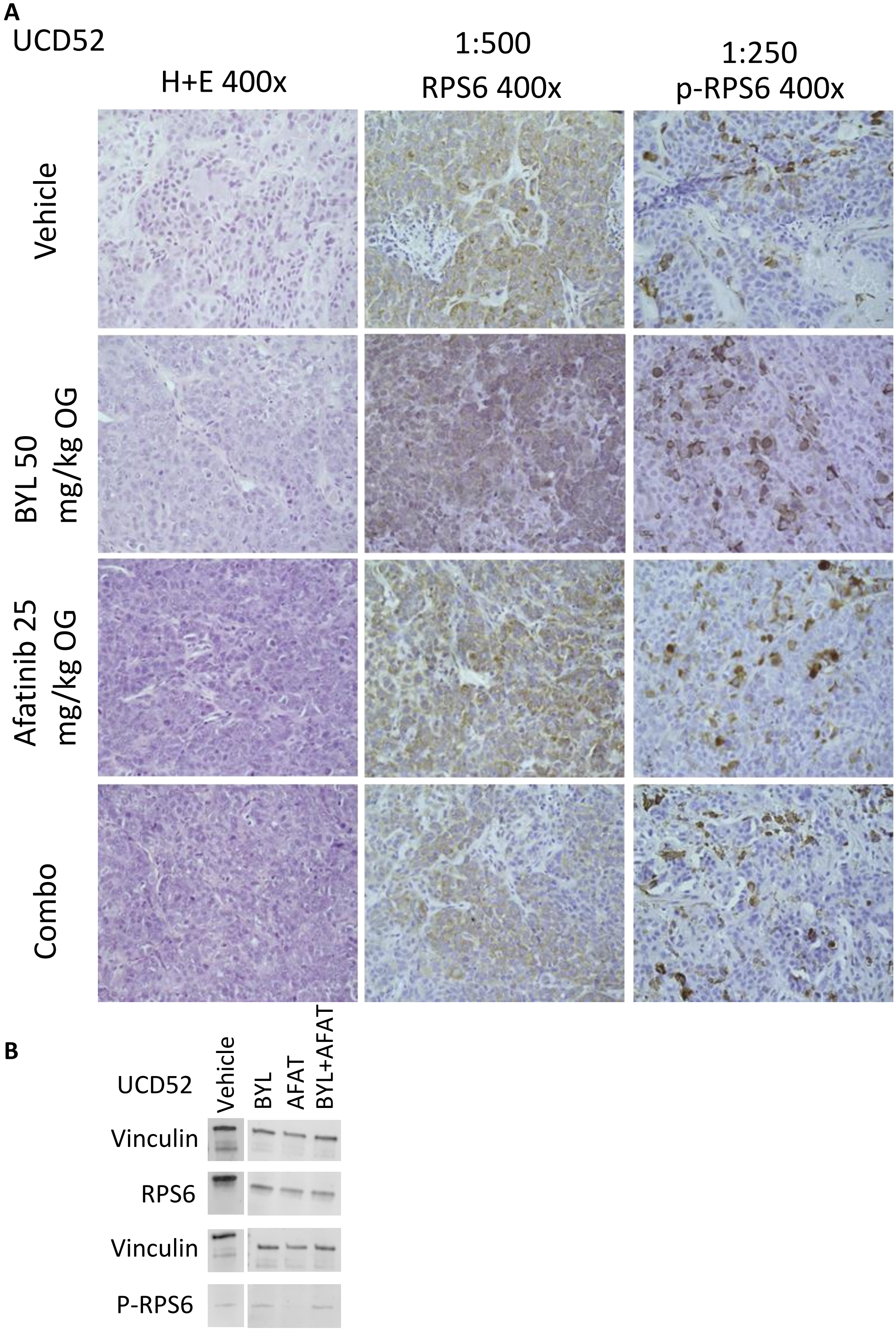

Supplemental Figure S5. Treatment did not show reduction in p-RPS6. (A) H+E and IHC of RPS6 and p-RPS6 of each treatment group in the BYL-719 with Afatinib trial in UCD52. (B) Western blots of Vinculin loading control and RPS6 and p-RPS6.

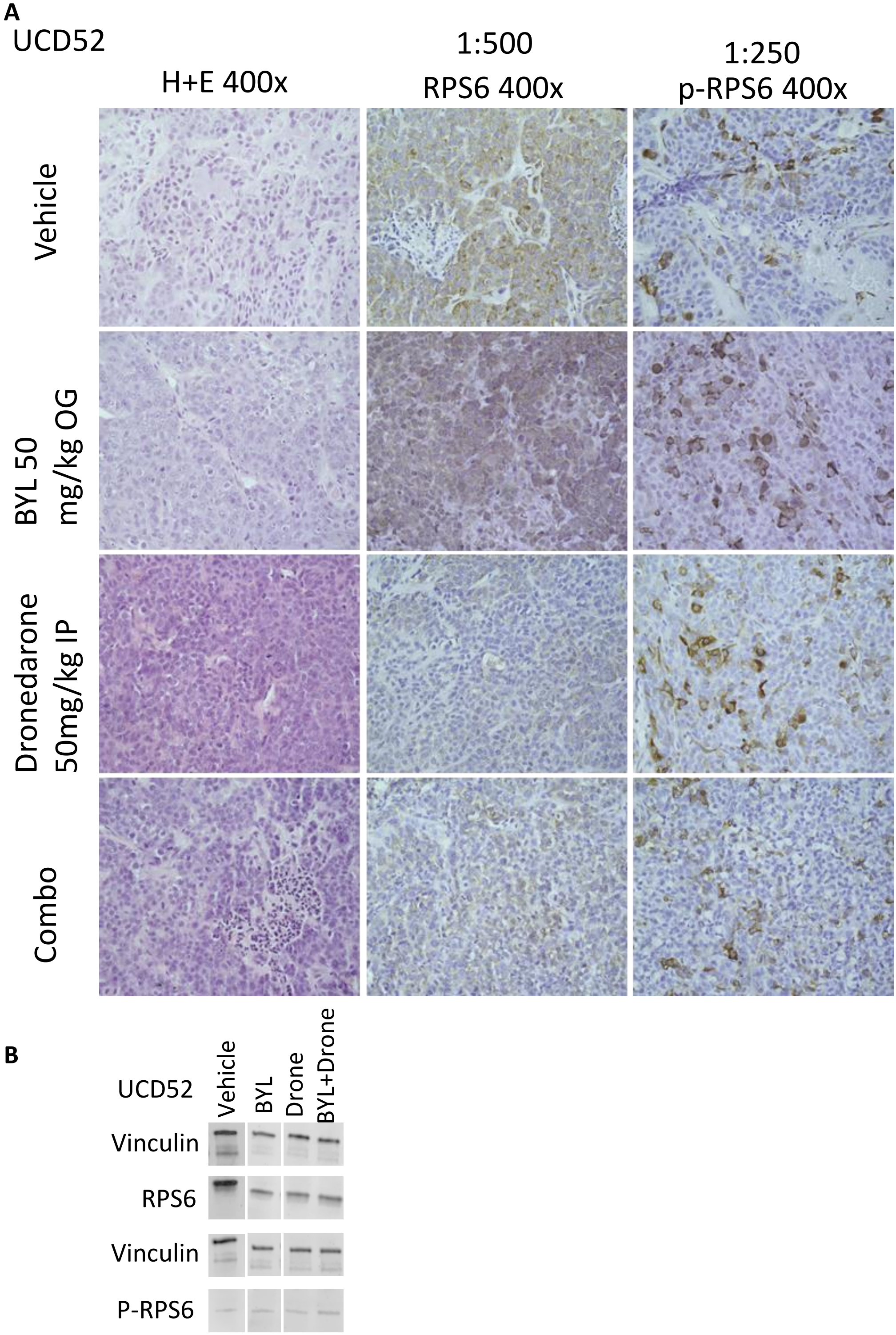

Supplemental Figure S6. Total RPS6 was reduced with dronedarone treatment in UCD52 in IHC but not in western. (A) H+E and IHC of RPS6 and p-RPS6 of each treatment group in the BYL-719 with Dronedarone trial in UCD52. (B) Western blots of Vinculin loading control and RPS6 and p-RPS6.

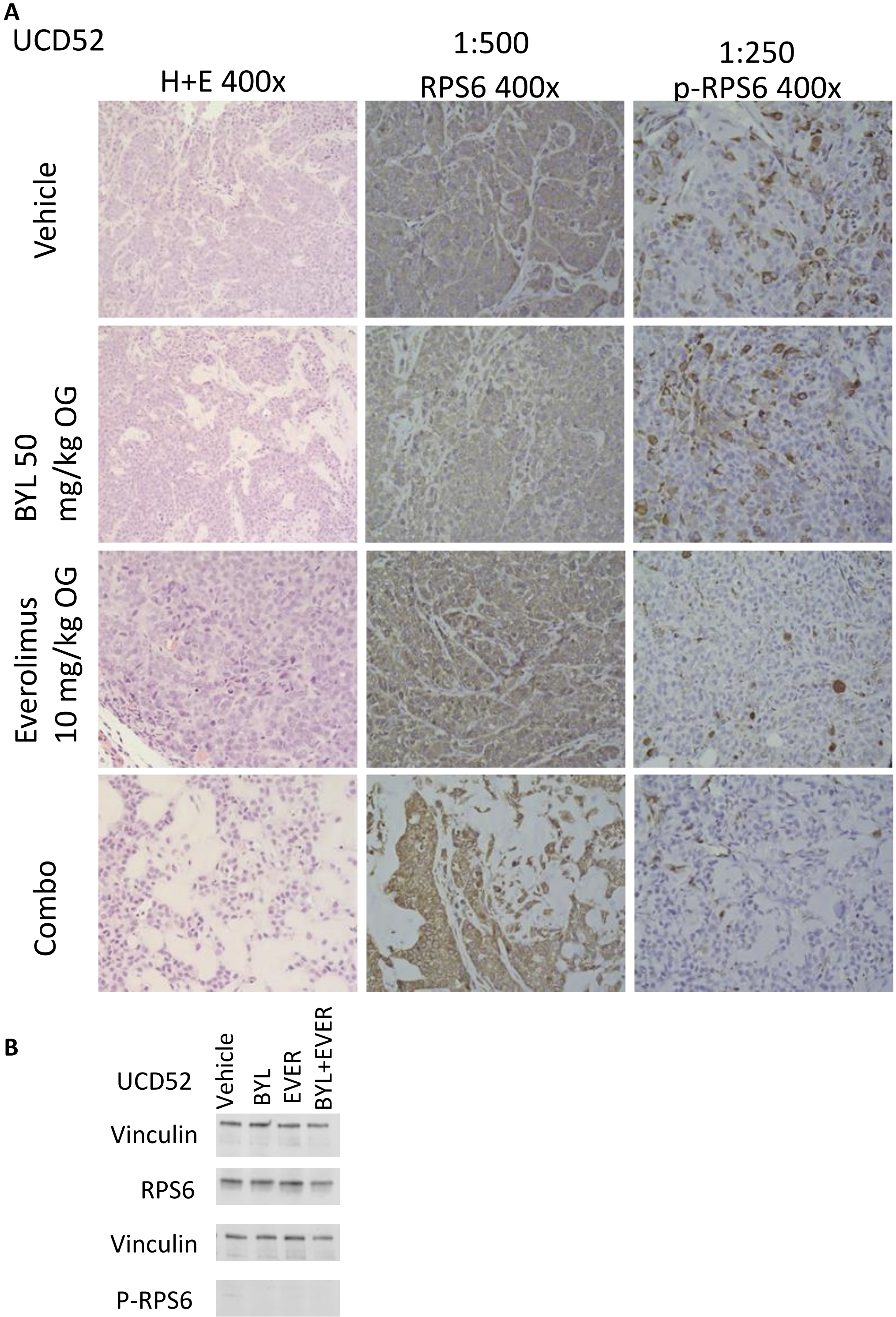

Supplemental Figure S7. The levels of p-RPS6 were reduced with treatment of either BYL-719 or Everolimus, and even further with combination therapy in UCD52. (A) H+E and IHC of RPS6 and p-RPS6 of each treatment group in the BYL-719 with Everolimus trial in UCD52. (B) Western blots of Vinculin loading control and RPS6 and p-RPS6.

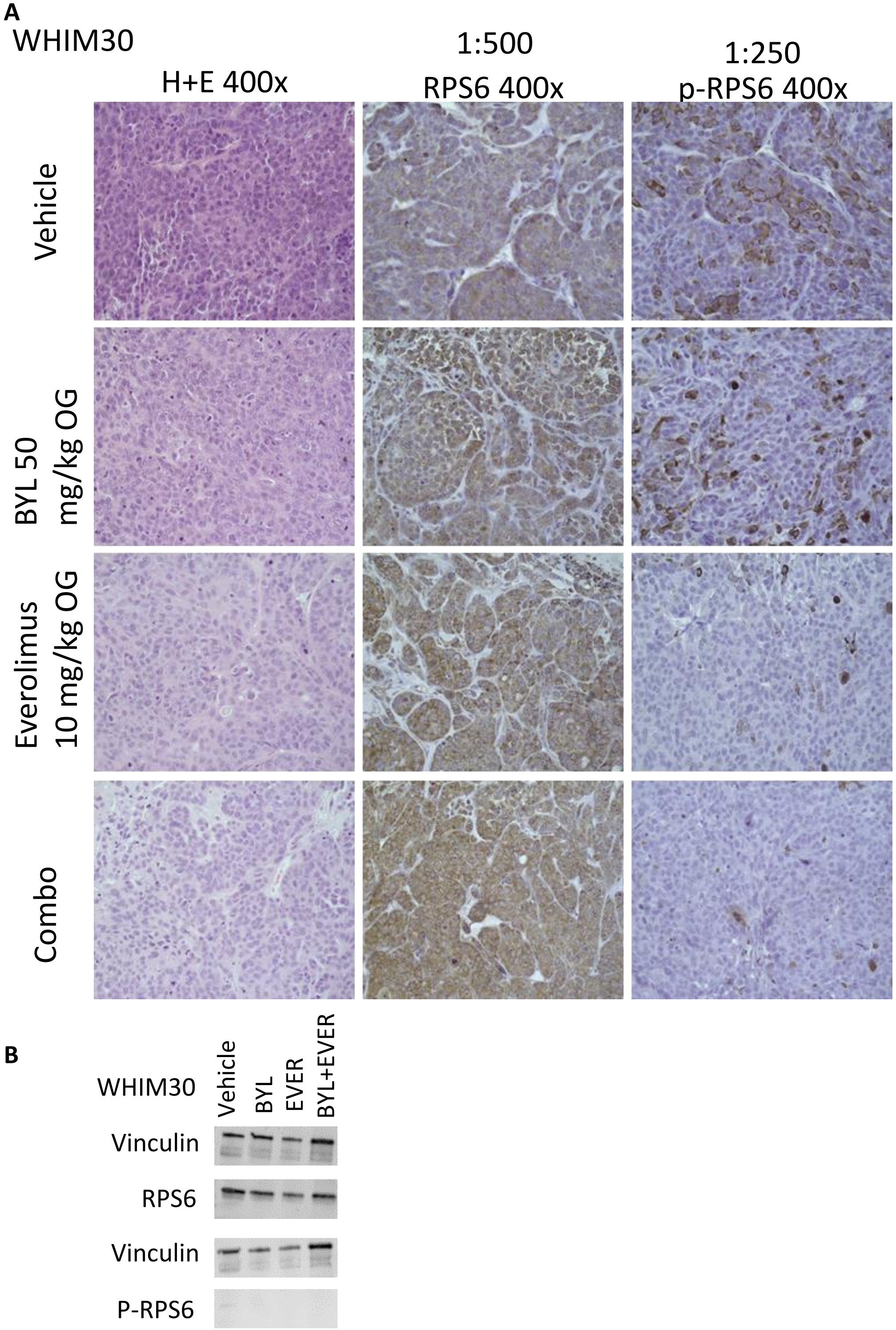

Supplemental Figure S8. The levels of p-RPS6 were reduced with treatment of either BYL-719 or Everolimus, and even further with combination therapy in WHIM30. (A) H+E and IHC of RPS6 and p-RPS6 of each treatment group in the BYL-719 with Everolimus trial in WHIM30. (B) Western blots of Vinculin loading control and RPS6 and p-RPS6.

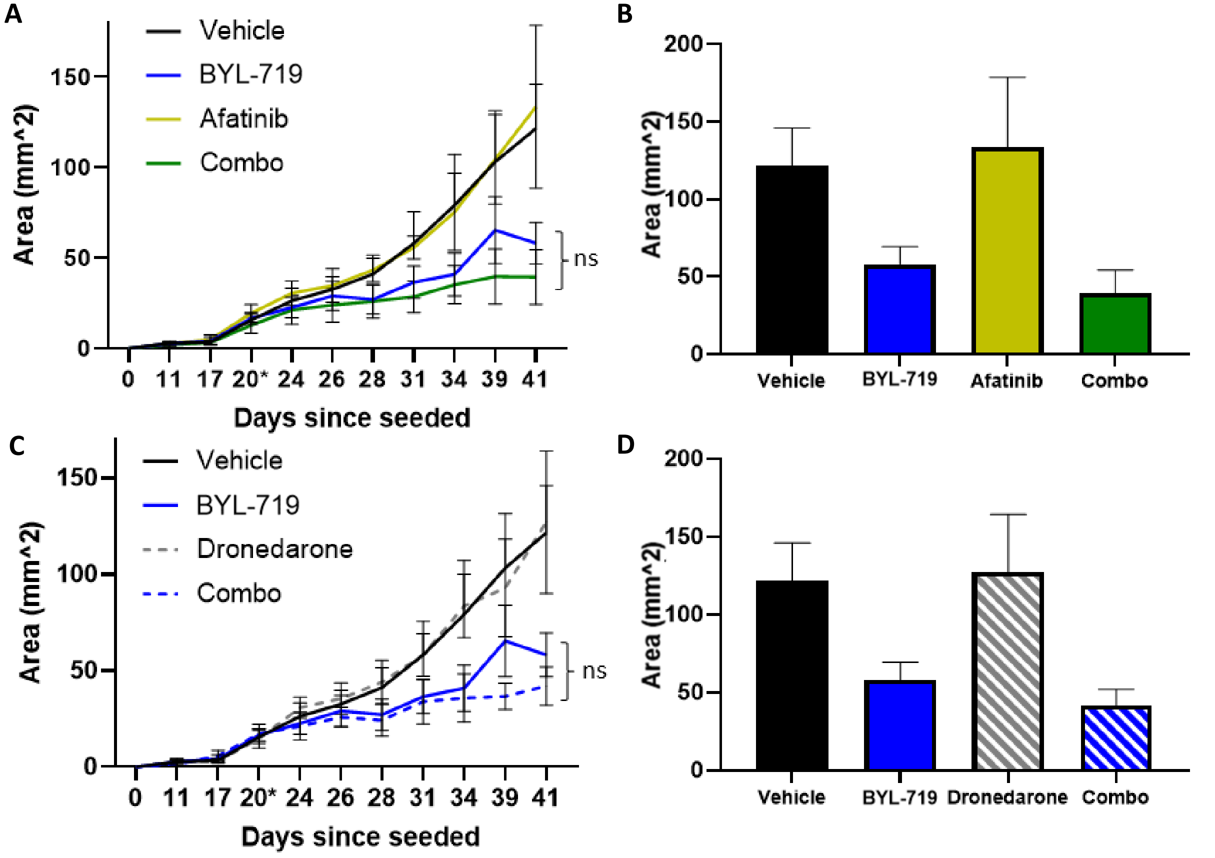

Supplemental Figure S9. Further combination therapies showed a trend of an effect in WHIM30. The final tumor area of the combinations of (A+B) Afatinib and (C+D) Dronedarone with BYL-719 was reduced relative to either treatment alone, but the effect of the combination was not significant relative to the effect of BYL-719 alone.

A

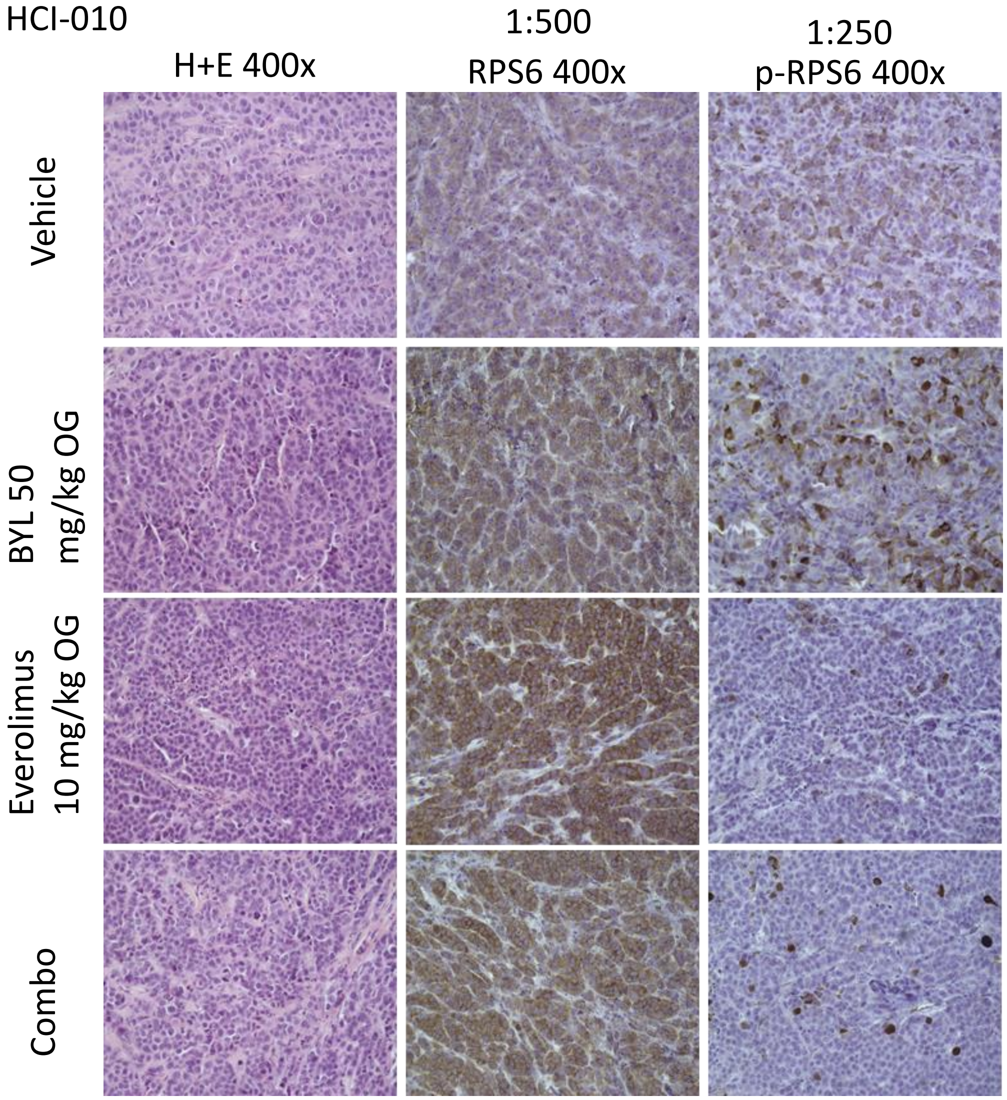

B

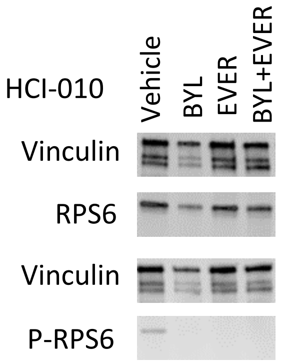

Supplemental Figure S10. The levels of p-RPS6 were reduced with treatment of Everolimus in HCI-010. (A) H+E and IHC of RPS6 and p-RPS6 of each treatment group in the BYL-719 with Everolimus trial in HCI-010. (B) Western blots of Vinculin loading control and RPS6 and p-RPS6.

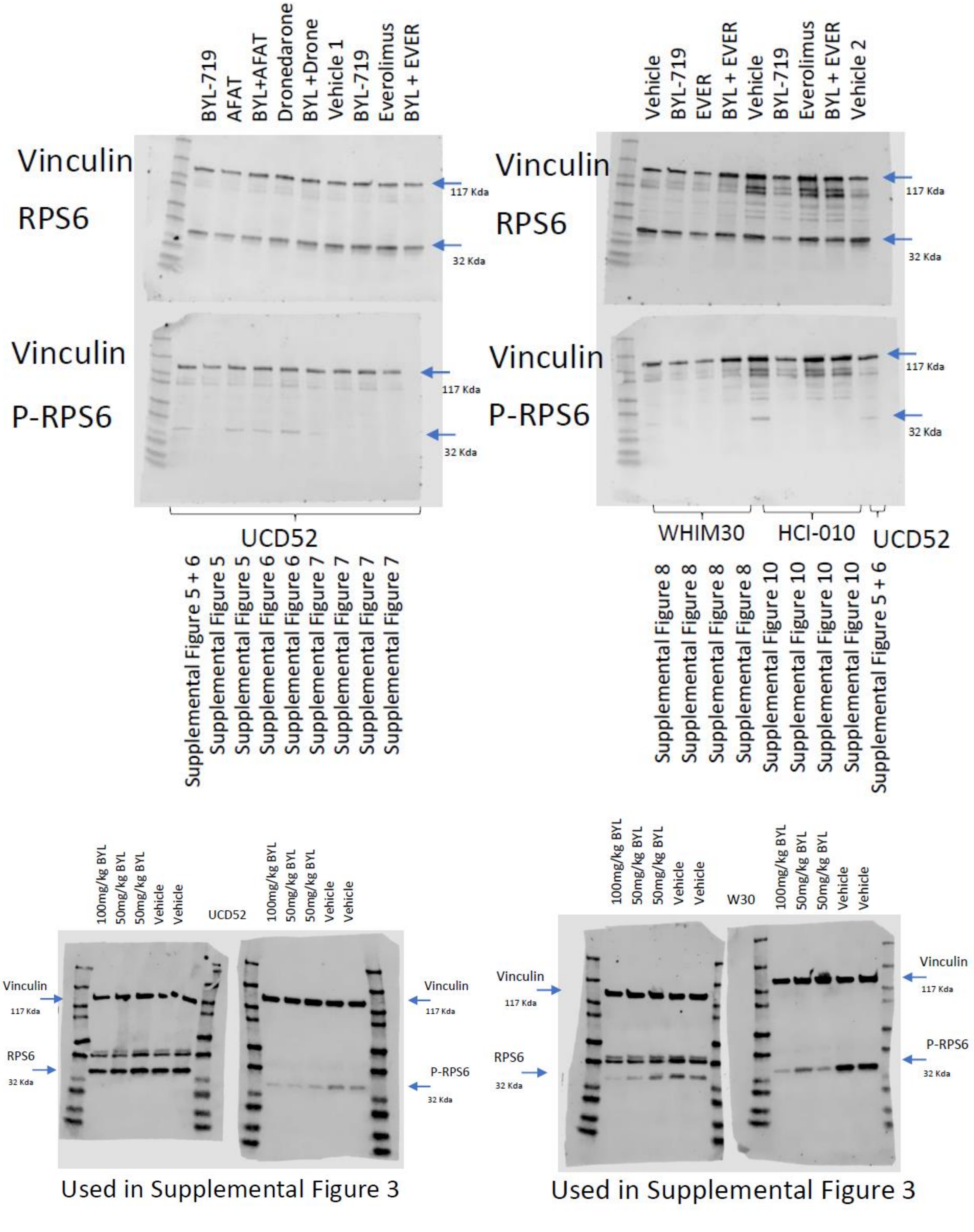

Supplemental Figure S11. Complete western blots of in vivo trials from tumors of the UCD52, WHIM30, and HCI-010 PDXs which were treated with vehicle, single agents, or combinations.

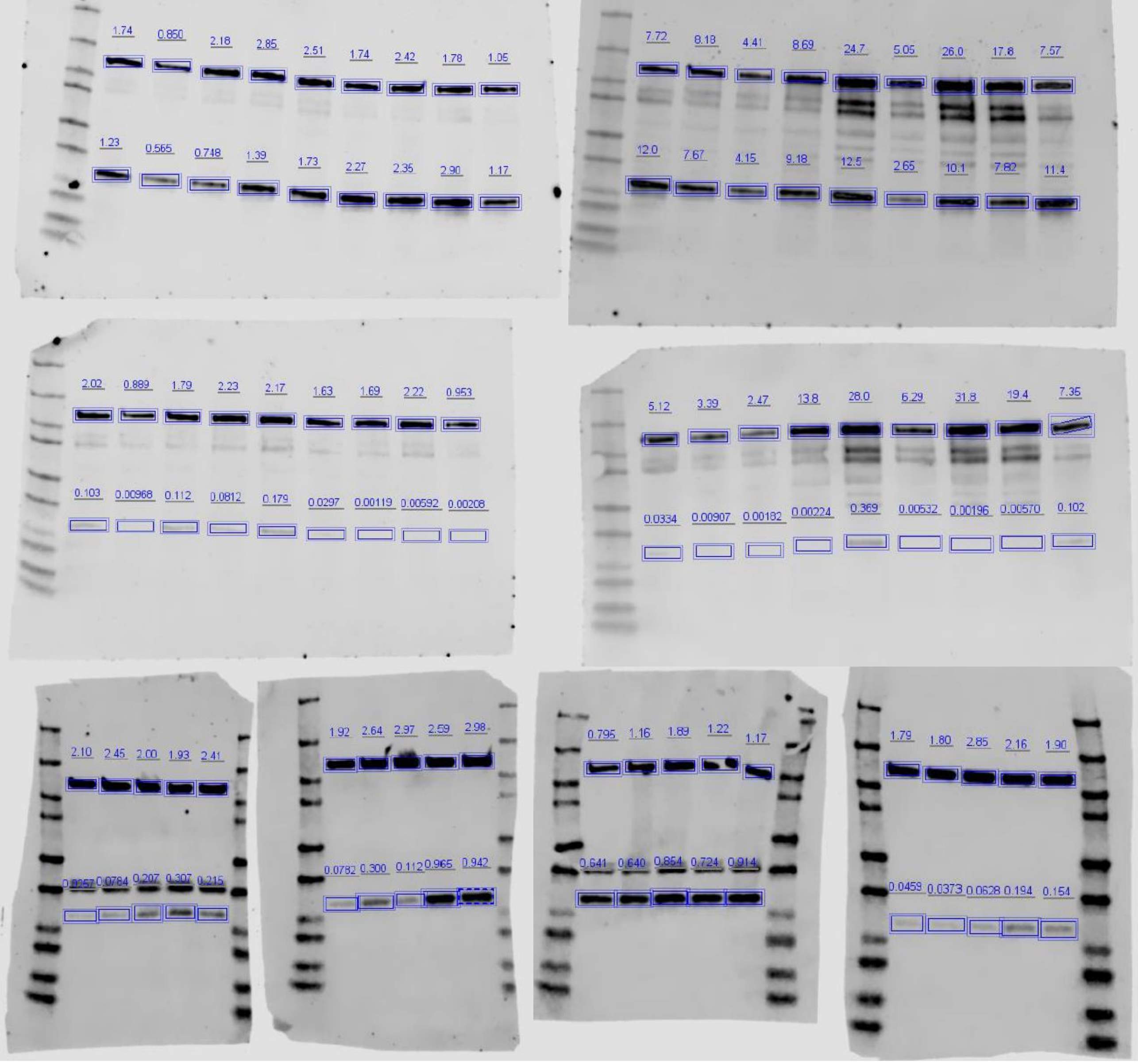

Supplemental Figure S12. Complete western blots of in vivo trials from tumors of the UCD52, WHIM30, and HCI-010 PDXs which were treated with vehicle, single agents, or combinations, values indicate densitometry.
